# miR-21 Targets ASPP2 to Inhibit Apoptosis via CHOP-Mediated Signaling in *Helicobacter pylori*-Infected Gastric Cancer Cells

**DOI:** 10.1155/2023/6675265

**Published:** 2023-07-28

**Authors:** Bo-Shih Huang, Chih-Ta Chen, Chao-Chi Yeh, Ting-Yu Fan, Fang-Yun Chen, Jyh-Ming Liou, Chia-Tung Shun, Ming-Shiang Wu, Lu-Ping Chow

**Affiliations:** ^1^Graduate Institute of Biochemistry and Molecular Biology, College of Medicine, National Taiwan University, Taipei, Taiwan; ^2^Department of Internal Medicine, National Taiwan University Hospital, Taipei, Taiwan; ^3^Department of Pathology, National Taiwan University Hospital, Taipei, Taiwan

## Abstract

*Helicobacte*r *pylori* (*H. pylori*) infection affects cell survival pathways, including apoptosis and proliferation in host cells, and disruption of this balance is the key event in the development of *H. pylori*-induced gastric cancer (HPGC). *H. pylori* infection induces alterations in microRNAs expression that may be involved in GC development. Bioinformatic analysis showed that microRNA-21 (miR-21) is significantly upregulated in HPGC. Furthermore, quantitative proteomics and in silico prediction were employed to identify potential targets of miR-21. Following functional enrichment and clustered interaction network analyses, five candidates of miR-21 targets, PDCD4, ASPP2, DAXX, PIK3R1, and MAP3K1, were found across three functional clusters in association with cell death and survival, cellular movement, and cellular growth and proliferation. ASPP2 is inhibited by *H. pylori*-induced miR-21 overexpression. Moreover, ASPP2 levels are inversely correlated with miR-21 levels in HPGC tumor tissues. Thus, ASPP2 was identified as a miR-21 target in HPGC. Here, we observed that *H. pylori*-induced ASPP2 suppression enhances resistance to apoptosis in GC cells using apoptosis assays. Using protein interaction network and coimmunoprecipitation assay, we identified CHOP as a direct mediator of the ASPP2 proapoptotic activity in *H. pylori*-infected GC cells. Mechanistically, ASPP2 suppression promotes p300-mediated CHOP degradation, in turn inhibiting CHOP-mediated transcription of Noxa, Bak, and suppression of Bcl-2 to enact antiapoptosis in the GC cells after *H. pylori* infection. Clinicopathological analysis revealed correlations between decreased ASPP2 expression and higher HPGC risk and poor prognosis. In summary, the discovery of *H. pylori*-induced antiapoptosis via miR-21-mediated suppression of ASPP2/CHOP-mediated signaling provides a novel perspective for developing HPGC management and treatment.

## 1. Introduction

Gastric cancer (GC) is ranked as the fifth most common malignancy worldwide [1]. In 2023, there were 26,500 newly diagnosed cases of GC and 11,130 deaths attributed to GC in the United States [2]. Furthermore, East Asia exhibits the highest incidence and mortality rates of GC [1]. The development of GC is influenced by various host factors, including gene polymorphisms, diabetes, and metabolic syndrome, and environmental factors, such as smoking, alcohol consumption, high-salt and nitrite-rich foods, EB virus infection [3], and notably, *Helicobacter pylori* (*H. pylori*) infection [4, 5]. *H. pylori* infection accounts for over 60% of the GC cases [6], and it is classified as a class I carcinogen, representing a significant etiologic factor in the development of GC.

*H. pylori* is a Gram-negative spiral-shaped bacterium that colonizes the human gastric mucosa, estimated to have infected approximately half the world's population. Chronic gastritis develops in virtually all persistent *H. pylori*-colonized individuals, among which the majority are asymptomatic and only 2–5% develop *H. pylori*-induced GC (HPGC) [7]. The most remarkable feature of persistent *H. pylori* infection is the chronic inflammatory response, which increases the risk of gastric malignancy [8, 9]. *H. pylori *infection-induced gastritis is mediated by multiple inflammatory cytokines [3]. The heterogeneity in *H. pylori *strain-specific virulence factors contributes to the variety of outcomes after infection [10]. The diverse outcomes of *H. pylori*-infected patients have additionally been attributed to different host responses [5].

*H. pylori*-induced inflammation disrupts the balance between cell proliferation and apoptosis in host cells, which represents a key event in the HPGC pathogenesis [11]. Apoptosis is regarded as a natural barrier to tumorigenesis and malignancy progression. Cancer cells engage various mechanisms to evade apoptosis that facilitate their survival. Several studies have reported that *H. pylori*-mediated antiapoptosis facilitates HPGC development [12–14]. For instance, *H. pylori* upregulates BIRC3 eRNA, which is essential for transcription of the apoptosis inhibitor, cIAP2, and confers resistance to apoptosis in the GC cells [12]. *H. pylori*-mediated DARPP-32 overexpression plays key roles in EGFR stabilization and AKT activation, in turn, preventing apoptosis in the GC cells [13]. Moreover, *H. pylori*-induced VCP transactivation is involved in proteasome degradation of various cellular factors and promotes the antiapoptotic response in the GC cells [14].

MiRNAs are small noncoding RNAs that regulate the expression of target genes through translational repression or mRNA degradation [15]. Accumulating evidence supports critical functions of miRNAs in carcinogenesis [16]. *H. pylori* infection induces alterations in expression of miRNAs that may be involved in GC development, including miR-21 [17], miR-584 [18], miR-222 [19], miR-223 [20], miR-181b [21], miR-27a [22], miR-320 [23], miR-101 [24], and miR-218 [25], which promote antiapoptosis and proliferation [26]. Among these, miR-21 has been identified in several tumors and is highly expressed in GC in particular, suggesting its involvement in regulation of tumor progression [27]. However, the precise functions and regulatory pathways of miR-21 target genes in gastric carcinogenesis remain obscure.

In the present study, miR-21 was identified as the most significantly upregulated miRNA in HPGC with the aid of bioinformatic approaches. miR-21 functions as an oncogenic miRNA involved in tumorigenesis by targeting tumor suppressive genes. For example, miR-21 downregulates PTEN to promote proliferation and antiapoptosis processes in acute myeloid leukemia cells [28]. Additionally, miR-21 targets PTEN, RECK, and Bcl-2 to enhance migration and invasion in lung cancer cells [29]. To elucidate the mechanisms underlying the involvement of miR-21 in HPGC pathogenesis, the stable isotope labeling with amino acids in cell culture (SILAC)-based quantitative proteomic approach was adopted to identify important downstream targets in the AGS cell model. Five potential miR-21-regulated target proteins were identified with different oncogenic functions in the GC cells. Among these, ASPP2 was identified as a miR-21 target in the HPGC. Subsequent experiments showed that ASPP2 downregulation induced by *H. pylori* infection promotes antiapoptosis through disruption of the CHOP-modulated pathway in the GC cells. Further clinical findings validated ASPP2 as a key determinant of HPGC pathogenesis. The collective results of this study provide novel insights into the mechanisms underlying HPGC malignancy. Moreover, the newly discovered prognostic potential of ASPP2 in HPGC progression may effectively assist in GC management and treatment.

## 2. Materials and Methods

### 2.1. Human Subjects and Tissue Specimens

In total, 790 patients with GC were enrolled from November 1998 to December 2012 in the National Taiwan University Hospital. Our study was approved by the Institutional Review Board of the Research Ethical Committee of the National Taiwan University Hospital (NTUH IRB number: 201903018RINA). Patient biopsies were reviewed by GI pathologists. *H. pylori* infection was detected via serologic enzyme-linked immunosorbent assay and histological analysis as described previously [4]. A total of 92 HPGC patients older than 20 years with surgical resection were included in this retrospective case-control study. Endoscopic biopsies of 89 non-GC subjects were additionally included (29 *H. pylori*-induced intestinal metaplasia (HPIM) cases, 30 *H. pylori*-induced gastritis (HPGS) cases, and 30 *H. pylori*-negative (HP (−)) healthy controls with no cancer in the digestive tract). Paraffin-embedded sections of HPGC tumors and paired adjacent normal and non-GC samples were prepared for further analyses. Demographic and clinicopathological features of the subjects were recorded for subsequent analyses. All procedures were conducted in accordance with the approved guidelines and regulations.

### 2.2. Bioinformatic Analysis

To identify potential dysregulated miRNAs in HPGC, we integrated data from two datasets. The first dataset, obtained from Gene Expression Omnibus (accession number: GSE62503), showed that 301 out of 718 miRNAs exhibited dysregulation in gastric tissues of HPGC compared to their counterparts in HPGS in a mouse model (*p* < 0.05). The second dataset, obtained from The Cancer Genome Atlas (data ID: TCGA.STAD.sampleMap/miRNA_HiSeq_gene), showed that 843 out of 2179 miRNAs displayed dysregulation in human GC tumors compared to adjacent normal control tissues (*p* < 0.05). Distribution of significance versus fold changes of a total of 80 common dysregulated miRNAs from the two datasets was further analyzed using the volcano plot. Among these miRNAs, miR-21 was the most significantly upregulated in HPGC (log_2_(fold change) >2.0 and -log (*p* value) >1.3). For the prediction of miR-21 targets, we utilized the miRSystem (http://mirsystem.cgm.ntu.edu.tw), which integrates seven different programs (DIANA-microT, miRanda, mirBridge, PicTa, PITA, rna22, and TargetScan), and conducted a BLAST search against two experimentally validated databases, TarBase and miRecords [30]. Biological function enrichment and clustered interaction network analyses of miR-21 candidate targets were performed via ingenuity pathway analysis (IPA, Ingenuity Systems, Redwood City, CA, USA; http://www.ingenuity.com). The ranking and significance of biological functions was based on the *p* values.

### 2.3. *In Situ* Hybridization and Immunohistochemistry (IHC)

Histological analysis of miR-21 was performed via *in situ* hybridization with a miRCURY LNA™ microRNA ISH Optimization Kit (Exiqon, Vedbæk, Denmark) as per the manufacturer's instructions. Briefly, sample slides were deparaffinized and stained with miR-21 probes. Cell nuclei were counterstained with Nuclear Fast Red™ (Thermo Fisher Scientific, Waltham, MA, USA). Expression of miR-21 was ranked based on H score ranging from 0 to 3, formulated as ((percentage of 0) × 0) + ((percentage of 1+) × 1) + ((percentage of 2+) × 2) + ((percentage of 3+) × 3) (0, nil; 1+, low or weak; 2+, moderate; and 3+, high or strong) [31]. For histological determination of ASPP2 and cleaved caspase 3 expression, IHC was performed with a Novocastra™ Manual Detection System kit (Leica Biosystems, Newcastle, UK) as described previously [31]. High expression was defined as positive staining in ≥50% of the cells.

### 2.4. Cell Culture, *H. pylori* Culture, and Infection

Human GC epithelial cell lines AGS (CRL-1739) and HEK293 (CRL-1573) were obtained from the American Type Culture Collection. Another GC epithelial cell line MKN45 was kindly provided by Professor Min-Chuan Huang from the Graduate Institute of Anatomy and Cell Biology, College of Medicine, National Taiwan University. AGS and HEK293 cells were maintained in Dulbecco's modified Eagle's medium (DMEM, HyClone, Marlborough, MA, USA) and MKN45 cells in Roswell Park Memorial Institute medium (HyClone) at 37°C in a humidified incubator under 5% CO_2_. Culture medium was supplemented with 10% fetal bovine serum (HyClone), penicillin (100 U/L), and streptomycin (10 mg/L). The *H. pylori* strain (ATCC 43504) was obtained from the American Type Culture Collection. *H. pylori* was inoculated on Columbia agar containing 5% sheep blood (BD Biosciences, San Jose, CA, USA) and grown at 37°C in a microaerophilic chamber under 10% CO_2_, 5% O_2_, and 85% N_2_. The study on *H. pylori* was approved by the Biosafety Committee of National Taiwan University. All procedures were conducted in compliance with the approved guidelines and regulations. AGS and MKN45 cells were infected with *H. pylori* at multiplicity of infection (MOI) of 30 and 90, respectively, for the indicated times after starvation in serum-free medium for 1.5 h.

### 2.5. Vector Construction, Transfection, and Viral Transduction

For adenovirus-based miR-21 overexpression, adenovirus particles with pAdTrack-CMV Mock vectors (AdMock) or vectors coding for primary miR-21 sequence (AdmiR-21) were prepared using the HEK 293 cells as described previously [14]. For adenoviral transduction, AGS cells were infected with adenovirus particles at MOI of 2 for 36 h to establish AdMock or AdmiR-21 AGS cells. MiR-21 overexpression efficiency was assessed with quantitative polymerase chain reaction (qPCR). For small nucleotide transfection or siRNA-mediated gene knockdown, 500 pmol control siRNA (siCtrl), miR-21, or anti-miR-21 oligonucleotide and siRNA against CHOP (siCHOP) or p300 (sip300, GenePharma, Shanghai, China) were transfected into AGS or MKN45 cells for 48 h with Lipofectamine (Thermo Fisher Scientific). For lentivirus-mediated ASPP2 knockdown, lentiviruses expressing control shRNA (shCtrl) or shRNA against ASPP2 (shASPP2) were prepared using HEK293 cells according to the supplier's instructions (RNAiCore, Academia Sinica, Taipei, Taiwan). For lentiviral transduction, medium containing shCtrl or shASPP2 virus was incubated with AGS and MKN45 cells for 72 h and the efficiency of ASPP2 knockdown was confirmed via qPCR and immunoblotting. For ASPP2 overexpression, the ASPP2 gene was cloned into p3xFLAG-CMV-14 vector using a cDNA library generated from AGS cells. Mock control or ASPP2-overexpressing vectors (5 *μ*g) were transfected into AGS and MKN45 cells with Lipofectamine for 48 h and the efficiency of ASPP2 overexpression was confirmed via qPCR and immunoblotting. The small nucleotide, shRNA, and siRNA target sequences are listed in Supplementary Table 1.

### 2.6. SILAC-Based Quantitative Proteomic Analysis

AdMock and AdmiR-21 AGS cells were labeled by culturing in light DMEM containing unlabeled amino acids or heavy DMEM containing [^13^C_6_]-L-lysine and [^13^C_6_, ^15^N_4_]-L-arginine (Invitrogen, Carlsbad, CA, USA), respectively. Cells were harvested and equal amounts of labeled protein extracts were mixed, reduced with 5 mM dithiothreitol and alkylated with 10 mM iodoacetamide. Denatured protein samples were digested with trypsin (1 : 100, w/w; Promega, Madison, WI, USA) at 37°C overnight and desalted. Labeled peptides were reconstituted and injected into a basic C18 column (Zorbax, 300 Extend-C18, 5 *μ*m, 4.6 × 150 mm; Agilent, Santa Clara, CA, USA) and fractionated into 24 fractions using a mobile phase of continuous acetonitrile gradient with 10 mM ammonia bicarbonate (pH 10.0). Each collected fraction was reconstituted, trapped on a reverse phase C18 guard column (Acclaim PepMap100, 3 *μ*m, 100 Å, 75 *μ*m × 2 cm; Thermo Fisher Scientific), and separated using a coupled reverse phase C18 chromatography column (Acclaim PepMap RSLC, 2 *μ*m, 100 Å, 75 *μ*m × 15 cm; Thermo Fisher Scientific) with an acetonitrile gradient in 0.1% formic acid at a flow rate of 250 nL/min. Full-scan mass spectra (m/z 300–1600) were acquired on an LTQ-Orbitrap Velos mass analyzer (Thermo Fisher Scientific) at a constant resolution power of 60,000 (target m/z versus delta m/z) within the scan-range. The lock mass was set at 445.12003 (polycyclodimethylsiloxane). Acquired spectra were processed, analyzed, and quantified using Proteome Discoverer software (version 1.3; Thermo Fisher Scientific) with the Mascot search engine (version 2.3.02) against the Swiss-Prot* Homo sapiens* protein database (version 57.2).

### 2.7. qPCR, Luciferase Reporter Assay, and Chromatin Immunoprecipitation (ChIP) Assays

In the qPCR assay, the total RNA in cell lysates or plasma specimens was isolated with TRIzol reagent (Thermo Fisher Scientific) and reverse-transcribed using a TaqMan MicroRNA Reverse Transcription Kit (Thermo Fisher Scientific). qPCR was conducted using a SensiFastTM SYBR Hi-ROX kit (BIOLINE, London, UK) and the transcripts were detected with the StepOne Plus system (Applied Biosystems, Cheshire, UK). Gene expression levels were calculated via the comparative 2^−△△CT^ method. In the luciferase reporter assay, AGS cells were transfected with control or miR-21 expression vector and pmirGLO vectors expressing wild-type or mutant ASPP2 3′UTR as indicated. The binding interactions between miR-21 and ASPP2 3′ UTR were evaluated at 24 h post-transfection using the Dual-Luciferase reporter gene kit (Promega). In the ChIP assay, AGS cell lysates were harvested and DNA-protein complexes were immunoprecipitated with control IgG, p65, or CHOP antibodies bound on Protein G or A beads (G-Biosciences, St Louis, MO, USA) at 4°C overnight. Immunoprecipitated DNA was retrieved and analyzed for promoters of miR-21, Noxa, Bak, or Bcl-2 using qPCR. The conserved motif PuPuPuTGCAAT(A/C)CCC for CHOP binding to Noxa and Bak promoter [32] and C/EBP binding site for CHOP binding to suppress Bcl-2 transcription [33, 34] were searched using ensembl (http://www.ensembl.org/index.html). The primer sequences are listed in Supplementary Table 1.

### 2.8. Immunoblotting and Coimmunoprecipitation (Co-IP) Assays

In the immunoblot assay, cells were lysed and separated via sodium dodecyl sulfate polyacrylamide gel electrophoresis (SDS-PAGE) and transferred to PVDF membranes (Merck Millipore, Burlington, MA, USA). Immunoblotting was performed with the indicated antibodies (listed in Supplementary Table 1) as described previously [35]. In the Co-IP assay, AGS cell lysates were harvested and incubated with antibodies against ASPP2, CHOP, or p300 bound on Protein G or A beads (G-Biosciences) at 4°C overnight. Immunoprecipitated protein complex was eluted and analyzed via SDS-PAGE and immunoblotting.

### 2.9. Terminal Deoxynucleotidyl Transferase dUTP Nick End Labeling (TUNEL) and Annexin V/Propidium Iodide (PI) Staining Apoptosis Assays

In the TUNEL assay, apoptotic cells were determined by detecting DNA double strand breaks using the APO-BRDU™ kit (Enzo Life Sciences, Farmingdale, NY, USA) according to the manufacturer's instructions. For the Annexin V/PI assay, cells were collected and stained with FITC-conjugated antibodies against Annexin V (BioLegend, San Diego, CA, USA) and PI (BioLegend). The percentages of apoptotic cells in both TUNEL and Annexin V/PI staining assays were measured with a Cytomics FC500 flow cytometer (Beckman Coulter, Brea, CA, USA) as described previously [14].

### 2.10. Statistical Analysis

Student's *t*-test was used for the general evaluation of the experimental significance and quantified values expressed as mean ± SD. The differences in expression and correlations between ASPP2 and miR-21 or cleaved caspase 3 in HPGC tumors and control counterparts were examined with the Mann–Whitney test and Spearman correlation analysis. ASPP2 levels among HPGC, HPIM, HPGS, and healthy control groups were evaluated using the Mann–Whitney test. Univariate and multivariate logistic regression analyses were utilized to compute the odds ratios (ORs), and 95% confidence interval (CI) of demographic features, and ASPP2 levels for HPGC. Univariate and multivariate Cox's proportional hazard regression models were used to compute the hazard ratios (HRs), and 95% CI of demographic and clinicopathological features, and ASPP2 levels in HPGC patients. OS curves based on ASPP2 expression were constructed using the Kaplan–Meier method and compared with the log-rank test. All statistical evaluations were two-tailed, with *p* values <0.05 considered statistically significant. All statistical analyses were performed using IBM SPSS Statistics (version 22.0; Chicago, IL, USA).

## 3. Results

### 3.1. NF-*κ*B-Mediated miR-21 Upregulation Is Involved in HPGC

**Figure 1:**
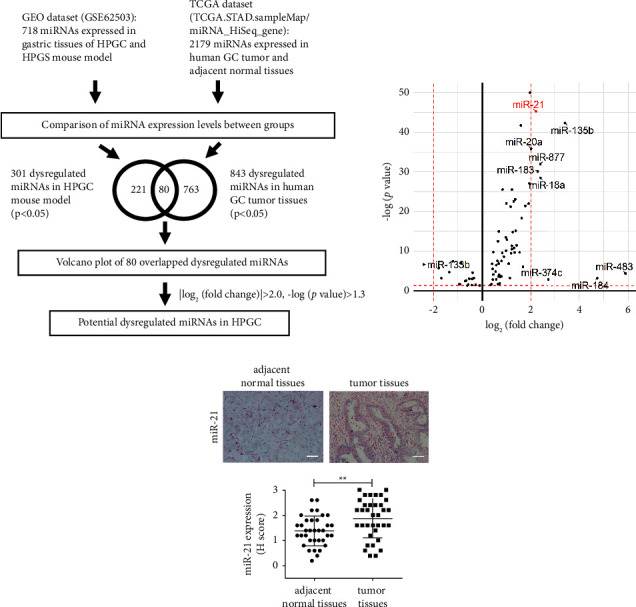
miR-21 is significantly overexpressed in HPGC. (a) Schematic description of the strategy to identify potentially upregulated miRNAs in HPGC. An integrated analysis of both GEO dataset (accession number: GSE62503) and TCGA datasets (data ID: TCGA.STAD.sampleMap/miRNA_HiSeq_gene) was performed, which resulted in the identification of 80 miRNAs that were commonly dysregulated. (b) Volcano plot showed miR-21 as a potentially upregulated miRNA in HPGC among 80 commonly dysregulated miRNA. (c) Relative miR-21 expression levels in HPGC tumor tissues (*n* = 35) as compared to adjacent normal tissues (*n* = 35) by *in situ* hybridization. Upper, representative images were captured at 200x magnification. Scale bar, 50 *μ*m. Lower, relative quantitation of miR-21 levels by H score ranking. The significance of differences in H scores between two groups was compared using the nonparametric Mann–Whitney *U* test. ^*∗∗*^*p* < 0.01.

*H. pylori* infection induces alterations in the expression of miRNAs that may be involved in the development of GC [26]. To clarify the involvement of miRNA dysregulation in HPGC pathogenesis, we aimed to identify potential miRNAs critical for HPGC using bioinformatic approaches. Using the GEO dataset, 301 significantly dysregulated miRNAs were identified in the gastric tissues of HPGC mice relative to their HPGS counterparts (*p* < 0.05). Eighty commonly dysregulated miRNAs were further extracted upon comparison with 843 significantly dysregulated miRNAs in human GC tumors (relative to paired adjacent normal controls, *p* < 0.05) from TCGA dataset (Figure 1(a)). The eighty miRNAs were further examined using a volcano scatter plot. Consequently, miR-21 was identified as the most significantly upregulated miRNA potentially associated with *H. pylori* infection in human GC (log_2_(fold change) >2.0 and −log (*p* value) >1.3; Figure 1(b)). Consistently, our results showed marked upregulation of miR-21 in HPGC tumor tissues (*n* = 35) compared to normal controls (*n* = 35; Figure 1(c)).

*In vitro, H. pylori* infection promoted miR-21 expression in AGS cells (Supplementary Figure 1A). The most remarkable feature of *H. pylori* infection is inflammatory response, which is mediated by the activation of NF-*κ*B [8, 9]. NF-*κ*B has been identified as a major transcriptional activator of miR-21 [36, 37]. The phosphorylation of the NF-*κ*B subunit p65 at Ser 536 facilitates the translocation of NF-*κ*B to the nucleus and initiates transcription, which is a crucial event for NF-*κ*B activation [38]. As shown in Supplementary Figure 1B, phosphorylation of p65 at Ser 536 in AGS cells increased after *H. pylori* infection, whereas treatment with MG132, a proteasome inhibitor of NF-*κ*B, led to significant suppression of p65 phosphorylation and miR-21 expression in *H. pylori*-infected AGS cells compared to nontreated controls (Supplementary Figures 1C and D). Moreover, the binding of p65 to miR-21 promoter was significantly increased in cells infected with *H. pylori* compared to noninfected controls (Supplementary Figure 1E). Collectively, these results suggest that *H. pylori*-induced miR-21 upregulation is mediated by NF-*κ*B activation in GC cells.

### 3.2. Identification of ASPP2 as a miR-21 Target in HPGC

**Figure 2:**
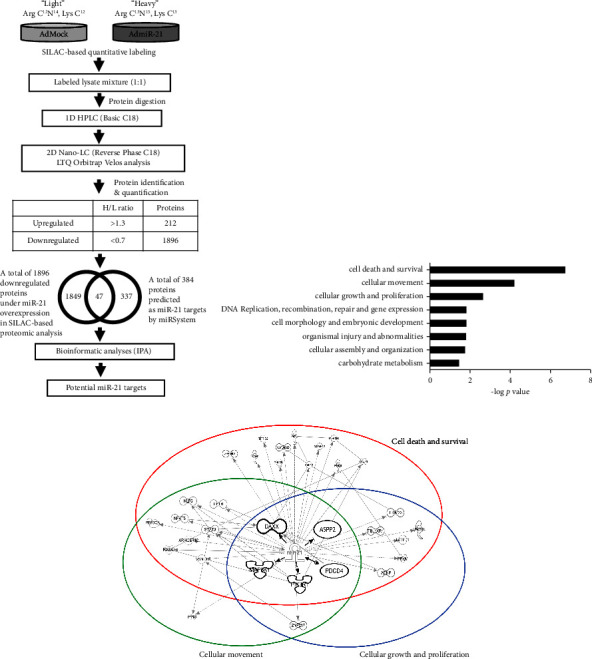
Quantitative proteomics reveals altered functional proteome in the GC cells after miR-21 overexpression. (a) Strategic flowchart for the identification of potential miR-21 targets. SILAC-labeled adenovirus-based mock and miR-21-overexpressing AGS cells (AdMock and AdmiR-21) were harvested. Proteins were extracted and digested with trypsin. Peptide mixture was fractionated via offline high-performance liquid chromatography and subsequently analyzed with an LTQ-Orbitrap Velos hybrid mass spectrometer. A total of 47 commonly downregulated proteins (AdmiR-21/AdMock ratio <0.7) were further extracted compared with predicted candidates in the miRsystem. Functional characterization of the 47 leads was performed via IPA to identify the potential miR-21 targets. (b) The eight functional groups that significantly enriched *p* value <0.05) in the 47 candidates of miR-21 targets, where cell death and survival, cellular movement, and cellular growth and proliferation were the top three enriched functions. (c) IPA interaction network analysis shows interacting linkage and functional clustering of molecules among the top three enriched functions. A central molecular hub across all three clusters was formed by the five candidates of miR-21 targets.

In view of the functional importance of miR-21 in GC, we further focused on the potential mechanisms underlying miR-21-mediated GC progression. We assessed the functional effects of miR-21 upregulation on GC cells with the aid of miR-21-overexpressing AGS cells. Upregulation of miR-21 clearly promoted proliferation, migration, and invasion and inhibited apoptosis of AGS cells (Supplementary Figures 2A–F). To further identify potential miR-21 targets that could contribute to the cancer phenotype, SILAC-based quantitative proteomic analysis and bioinformatic integration were performed. A schematic description of the approach is illustrated in Figure 2(a). Differential proteomes were compared between AGS cells infected with AdMock and AdmiR-21. A total of 47 downregulated candidate targets of miR-21 (AdmiR-21/AdMock ratio <0.7) were further identified via *in silico* validation by miRSystem.

To clarify the functional characteristics of the 47 candidate miR-21 targets, these proteins were subjected to the biological function analysis using IPA. We found eight main functional groups that strongly enriched (*p* value <0.05) in miR-21-regulated proteins (Figure 2(b)). Interestingly, we observed top three enriched functions, cell death and survival, cellular movement, and cellular growth and proliferation, corresponding to the results of miR-21 functional assays (Supplementary Figures 2A–2F). For identifying potential targets under miR-21 regulation during oncogenesis, the interacting linkage and functional clustering of molecules among the top three enriched biological functions were performed via IPA interaction network analysis. As shown in Figure 2(c) and Supplementary Table 3, we observed 32 nonredundant molecules involved in the formation of three interconnected functional clusters modulated by miR-21, including cell death and survival (*n* = 30), cellular movement (*n* = 15), and cellular growth and proliferation (*n* = 12). Among the miR-21-regulated functional network, a central molecular hub across all three clusters formed by five candidate miR-21 targets was noticed, including Programmed Cell Death 4 (PDCD4), Apoptosis-Stimulating of P53 Protein 2 (ASPP2), Death-Domain Association Protein (DAXX), Phosphoinositide-3-Kinase Regulatory Subunit 1 (PIK3R1), and Mitogen-Activated Protein Kinase Kinase Kinase 1 (MAP3K1).

**Figure 3:**
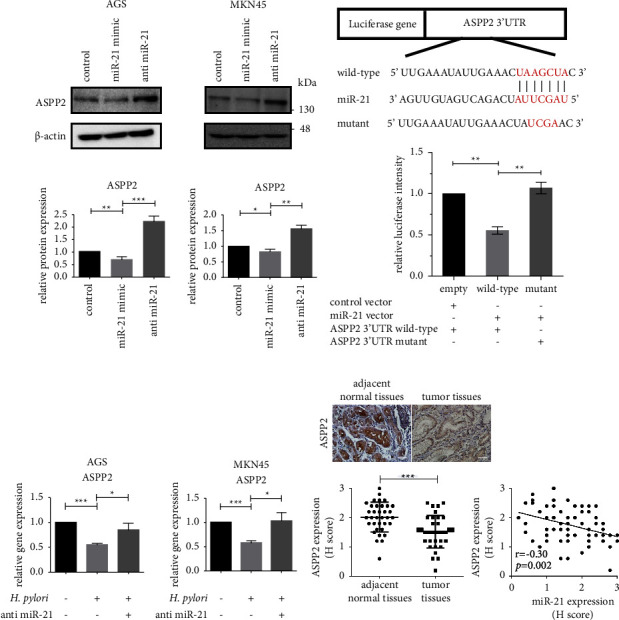
ASPP2 is a miR-21 target in HPGC. (a) AGS and MKN45 cells were transfected with control, miR-21 mimic, or anti-miR-21 oligonucleotides. ASPP2 protein levels in the cell lysates were analyzed via immunoblotting. (b) The binding sites between miR-21 and ASPP2 3′UTR were evaluated using TargetScan analysis (upper). Subsequently, AGS cells transfected with the indicated vectors and relative luciferase activity in AGS cells with wild-type or mutant ASPP2 3′UTR were analyzed by luciferase reporter assay (lower). (c) AGS and MKN45 cells transfected with control or anti-miR-21 oligonucleotides were incubated with *H. pylori* at 30 and 90 MOI or PBS for 6 hours. Relative ASPP2 mRNA levels were measured by qPCR. All the data were presented as mean ± SD of 3 replicates. (d) Relative ASPP2 expression levels in HPGC tumor tissues (*n* = 35) as compared to adjacent normal tissues (*n* = 35) by IHC and the correlation with miR-21 levels. Upper left, representative images were captured at 200x magnification. Scale bar, 50 *μ*m. Lower left, relative quantitation of ASPP2 levels by *H* score ranking. The significance of differences in *H* score between the two groups was compared using the nonparametric Mann–Whitney *U* test. Right, Spearman correlation analysis between miR-21 and ASPP2 levels in HPGC tumor tissues (*n* = 35). ^*∗*^*p* < 0.05, ^*∗∗*^*p* < 0.01, and ^*∗∗∗*^*p* < 0.001.

We further investigated the regulation of miR-21 on five candidate targets in GC cells using miR-21 overexpression and suppression assays. As shown in Supplementary Figure 3, we observed the most significant post-transcriptional regulation between miR-21 and ASPP2 in AGS cells, with PDCD4 serving as a positive control [39]. Therefore, ASPP2, playing key roles in modulation of cell growth and apoptosis and reported to exert tumor suppressor effects [40], was selected for further analysis. A quantitative mass spectrum of the representative peptide of ASPP2 was shown in Supplementary Figure 4. Moreover, protein levels of ASPP2 were consistently modulated by miR-21 in AGS and MKN45 cells (Figure 3(a)), further confirming the direct association between miR-21 and ASPP2. In the TargetScan analysis, miR-21 was predicted to bind the 3′UTR of ASPP2 (Figure 3(b), upper). The binding of miR-21 to the ASPP2 3′UTR was subsequently validated using the luciferase reporter assay. Overexpression of miR-21 significantly inhibited the luciferase reporter activity of the wild-type ASPP2 3′UTR, but not the mutant ASPP2 3′UTR. This provides evidence that ASPP2 is a direct target of miR-21 in the GC cells (Figure 3(b), lower).

We additionally examined ASPP2 expression under *H. pylori*-induced miR-21 overexpression in both GC cells. ASPP2 was downregulated in AGS and MKN45 cells after *H. pylori* infection for 6 h, while it was recovered under miR-21 inhibition by the treatment of anti-miR-21 (Figure 3(c)). The results suggest that *H. pylori*-induced ASPP2 suppression is mediated by miR-21. To validate the clinical significance of ASPP2 in HPGC, we analyzed its expression in tumor and surrounding normal tissues using IHC. As shown in Figure 3(d), left, ASPP2 expression was significantly lower in HPGC tumor tissues (*n* = 35) compared to adjacent normal counterparts (*n* = 35). Moreover, the evaluation of ASPP2 and miR-21 levels indicated a negative correlation in HPGC (Figure 3(d), right). Our data collectively indicate that ASPP2 is a miR-21 target in HPGC.

### 3.3. *H. pylori*-Induced ASPP2 Suppression Promotes Antiapoptosis of HPGC

In view of the implication of miR-21 upregulation in association with antiapoptosis from functional assays and proteomic analyses, we next clarify the antiapoptotic role of ASPP2 suppression in GC cells. shASPP2 and shCtrl lentivirus were generated for infection of AGS and MKN45 cells. ASPP2 mRNA and protein levels were efficiently suppressed in both shASPP2 GC cells compared to shCtrl cells (Supplementary Figure 5A). Results from TUNEL and Annexin V/PI assays indicated that knockdown of ASPP2 attenuated 5-fluorouracil (5-FU)-induced apoptosis in both the AGS and MKN45 cells, providing evidence that ASPP2 plays a role in apoptosis in GC cells (Supplementary Figures 5B and C).

**Figure 4:**
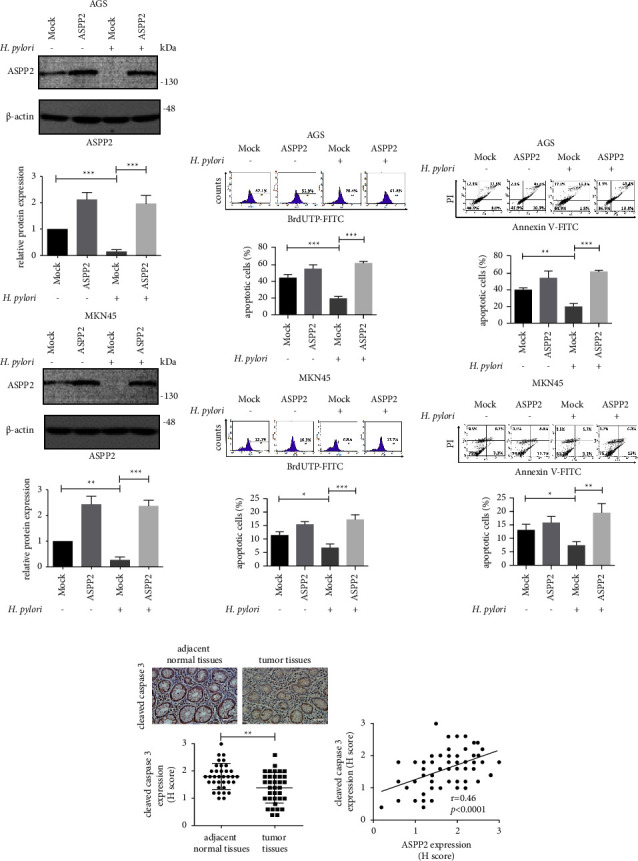
*H. pylori* induces ASPP2 downregulation and inhibition of apoptosis in HPGC. (a–c) AGS and MKN45 cells transfected with mock and ASPP2-overexpressing vectors were incubated with *H. pylori* at 30 and 90 MOI or PBS for 32 h. (a) ASPP2 protein levels in the cell lysates were measured via immunoblotting. (b) The percentage of apoptotic cells was analyzed by TUNEL assay. (c) The percentage of early and late apoptotic cells was assessed via Annexin V/PI assay. All the data were presented as mean ± SD of 3 replicates. (d) Relative cleaved caspase 3 expression levels in HPGC tumor tissues (*n* = 35) as compared to adjacent normal tissues (*n* = 35) by IHC and the correlation with ASPP2 levels. Upper left, representative images were captured at 200x magnification. Scale bar, 50 *μ*m. Lower left, relative quantitation of cleaved caspase 3 levels by H score ranking. The significance of differences in H score between two groups was compared using the nonparametric Mann–Whitney *U* test. Right, Spearman correlation analysis between ASPP2 and cleaved caspase 3 levels in HPGC tumor tissues (*n* = 35). ^*∗*^*p* < 0.05, ^*∗∗*^*p* < 0.01, and ^*∗∗∗*^*p* < 0.001.

To study the role of *H. pylori-*induced ASPP2 suppression in the apoptosis of GC cells, we subsequently examined the effects of *H. pylori* infection on apoptosis by time-dependent experiments in AGS and MKN45 cells. As shown in Supplementary Figures 6A and B, after *H. pylori* infection in AGS and MKN45 cells, induction of apoptosis in the GC cells was observed from 8 to 24 h, whereas antiapoptosis was stimulated rapidly at 32 h post-infection. Next, we observed the effect of *H. pylori-*induced ASPP2 suppression on the apoptosis in GC cells. As shown in Figure 4(a), *H. pylori* induced ASPP2 suppression in AGS and MKN45 cells at 32 h post-infection, which was rescued following ectopic overexpression of ASPP2. Data from the TUNEL assay showed that *H. pylori*-induced ASPP2 downregulation inhibited apoptosis in mock GC cells, which was recovered under conditions of ASPP2 overexpression (Figure 4(b)). Consistently, Annexin V/PI assays confirmed induction of antiapoptosis through ASPP2 suppression in *H. pylori*-infected GC cells (Figure 4(c)). In clinical patient samples, apoptotic maker cleaved caspase 3 was significantly downregulated in HPGC tumor tissues (*n* = 35) compared to adjacent normal counterparts (*n* = 35, Figure 4(d), left). Correlation analysis additionally indicated a positive correlation of ASPP2 attenuation with cleaved caspase 3 downregulation in HPGC tumors (Figure 4(d), right). These findings support a key antiapoptotic role of *H. pylori*-induced ASPP2 suppression in the progression of HPGC.

### 3.4. ASPP2 Interaction with CHOP Regulates the Expression of CHOP-Modulated Apoptotic Factors in *H. pylori*-Infected GC Cells

**Figure 5:**
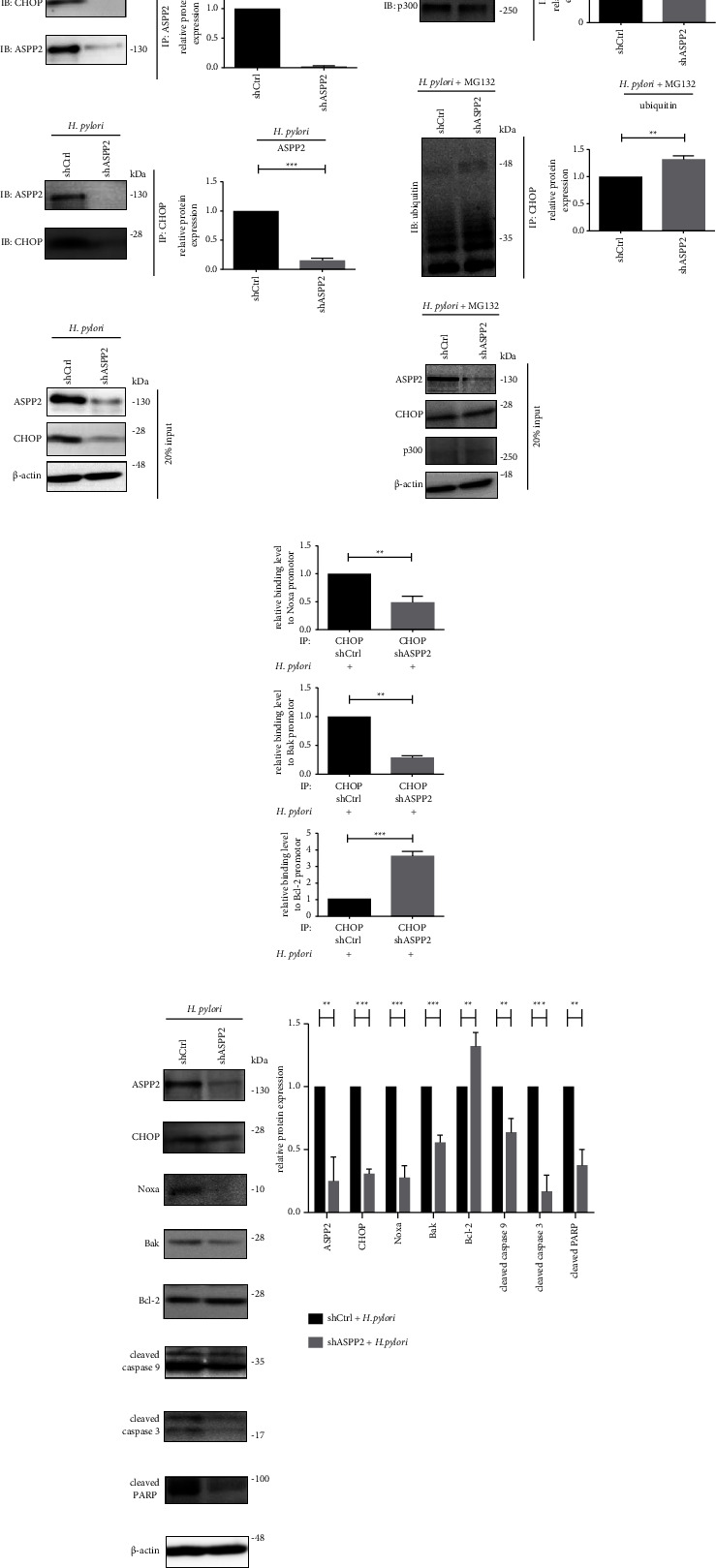
ASPP2 suppression promotes p300-mediated CHOP degradation and inhibits CHOP-mediated apoptotic signaling in the *H. pylori*-infected GC cells. (a) Cell lysates of shCtrl and shASPP2 AGS cells incubated with *H. pylori* at 30 MOI for 8 h were incubated with antibodies against ASPP2 (upper) or CHOP (middle) immobilized on protein G beads for 16 hours. (b) Cell lysates of shCtrl and shASPP2 AGS cells incubated with *H. pylori* at 30 MOI for 8 h followed by 10 *μ*M MG132 for 3 h were immunoprecipitated with antibodies against p300 (upper) or CHOP (middle) immobilized on protein A beads for 16 hours. In panels (a) and (b), the Co-IP complex and 20% input (lower) sample were subjected to immunoblotting. (c) Chromatin of shCtrl and shASPP2 AGS cells incubated with *H. pylori* at 30 MOI for 8 h were immunoprecipitated with CHOP antibodies immobilized on protein G or A beads for 16 hours. ChIP complex was retrieved and analyzed for relative CHOP binding level to promoters of Noxa (upper), Bak (middle), and Bcl-2 (lower) using qPCR. (d) shCtrl and shASPP2 AGS cells were incubated with *H. pylori* at 30 MOI for 8 h. Cell lysates were analyzed by immunoblotting using the indicated antibodies. All the data were presented as mean ± SD of 3 replicates. ^*∗∗*^*p* < 0.01 and ^*∗∗∗*^*p* < 0.01.

ASPP2 is involved in apoptosis through interactions with a variety of apoptosis-associated proteins [40]. To elucidate the mechanisms underlying ASPP2 suppression-mediated antiapoptosis in HPGC, an IPA mapping tool was utilized to identify key downstream molecules of ASPP2 by constructing a protein interaction network incorporating potential ASPP2 interactants and apoptosis-associated proteins. As shown in Supplementary Figure 7A, CHOP was identified as a highly connected molecule within the network. The significance of CHOP as a potential downstream mediator of ASPP2-modulated apoptosis was further studied. In TUNEL and Annexin V/PI analyses, knockdown of CHOP induced significant inhibition of *H. pylori*-induced apoptosis in AGS cells relative to control counterparts (Supplementary Figure 7B). Co-IP assay was performed to detect the formation of ASPP2/CHOP complex in AGS cells infected with *H. pylori*. As shown in Figure 5(a), CHOP was pulled down with the ASPP2 Co-IP complex in AGS cells after *H. pylori* infection for 8 h. Reciprocal Co-IP experiment confirmed direct interactions between ASPP2 and CHOP. We also detected that knockdown of ASPP2 could decrease expression of CHOP in *H. pylori*-infected AGS cells, suggesting that ASPP2 is involved in regulation of CHOP degradation. Earlier reports suggest that p300 E4 ligase is a crucial factor in degradation of CHOP [41]. Knockdown of p300 suppressed CHOP ubiquitination and enhanced its protein expression in AGS cells after *H. pylori* infection for 8 h, suggesting p300 modulates the ubiquitination and degradation of CHOP (Supplementary Figure 7c). We further investigated the role of ASPP2 in p300-mediated CHOP degradation. As shown in Figure 5(b), ASPP2 knockdown under treatment with MG132 promoted interactions of CHOP with p300, leading to increase in CHOP ubiquitination in AGS cells after *H. pylori* infection for 8 h, indicating the importance of ASPP2 in the regulation of CHOP/p300 interaction as well as CHOP activity under *H. pylori* infection.

CHOP was proposed to act as a transcription factor potentially regulating the expression of both proapoptotic (Noxa, Bak, Bim, Bax, PUMA, and DR5) and antiapoptotic (Bcl-2, Bcl-xL, and Mcl-1) genes [42, 43]. After screening several CHOP-regulated transcription factors, knockdown of CHOP significantly reduced Noxa and Bak, while enhanced Bcl-2 at the transcription level in AGS cells after *H. pylori* infection for 8 h (Supplementary Figure 7D). We further examined the role of ASPP2 in the regulation of CHOP-mediated transcription of apoptotic factors using ChIP assay. As shown in Figure 5(c), ASPP2 knockdown led to reduced binding of Noxa and Bak and enhanced binding of Bcl-2 promoter regions by CHOP in AGS cells after *H. pylori* infection for 8h. Additionally, ASPP2 knockdown decreased CHOP, Noxa, and Bak and increased Bcl-2 protein levels in *H. pylori*-infected AGS cells, where the expressions of cleaved caspase 9, caspase 3, and PARP were also attenuated (Figure 5(d)). Taken together, interaction with ASPP2 prevents p300-mediated degradation of CHOP, in turn promoting transcription of Noxa, Bak, and suppression of Bcl-2 expression in GC cells after *H. pylori* infection.

### 3.5. H. pylori-Induced ASPP2 Downregulation Promotes Antiapoptosis through Attenuation of CHOP-Modulated Apoptotic Pathway in GC Cells

**Figure 6:**
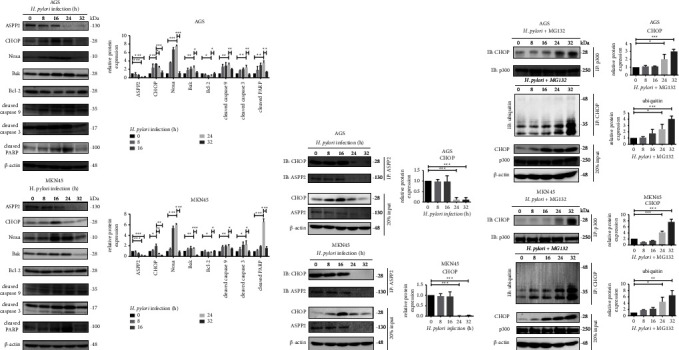
*H. pylori*-induced ASPP2 suppression promotes p300-mediated CHOP degradation and inhibits CHOP-mediated apoptotic signaling in the GC cells. (a) AGS and MKN45 cells were incubated with *H. pylori* at 30 and 90 MOI for the indicated time. Cell lysates were analyzed by immunoblotting using the indicated antibodies. (b) Cell lysates of AGS and MKN45 cells incubated with *H. pylori* at 30 and 90 MOI for the indicated time were immunoprecipitated with antibodies against ASPP2 (upper) immobilized on protein G beads for 16 hours. (c) Cell lysates of AGS and MKN45 cells incubated with *H. pylori* at 30 and 90 MOI for the indicated time followed by 10 *μ*M MG132 for 3 h were immunoprecipitated with antibodies against p300 (upper) or CHOP (middle) immobilized on protein A beads for 16 hours. In panels (b) and (c), the Co-IP complex and 20% input (lower) samples were subjected to immunoblotting. All the data were presented as mean ± SD of 3 replicates. ^*∗*^*p* < 0.05, ^*∗∗*^*p* < 0.01, and ^*∗∗∗*^*p* < 0.01.

Next, we examined the effect of *H. pylori* infection on the molecular changes of ASPP2/CHOP-mediated apoptotic pathway in a time-dependent manner. As shown in Figure 6(a), *H. pylori* induced a time-dependent increase in Noxa, Bak, cleaved caspase 9, caspase 3, and PARP, while simultaneously reducing Bcl-2 levels in AGS and MKN45 cells for up to 24 h post-infection. At 24 h post-infection, *H. pylori* induced downregulation of ASPP2 (Figure 6(a)) and a concomitant decrease in the interaction between ASPP2 and CHOP (Figure 6(b)) in AGS and MKN45 cells. Under the treatment with MG132, *H. pylori* enhanced interaction between CHOP and p300, which led to CHOP ubiquitination (Figure 6(c)) at 24 h post-infection in both GC cells. Interestingly, *H. pylori*-induced CHOP degradation after 24 h quickly reversed the expression of the apoptotic factors at 32 h post-infection, indicating a rapid shift towards antiapoptosis in the GC cells (Figure 6(a)). Consistent with the results in Supplementary Figure 6A and B, increase of apoptosis was observed in a time-dependent manner within 24 h of infection whereas antiapoptotic effect was rapidly stimulated at 32 h post-infection. These findings support a crucial role of ASPP2 suppression in *H. pylori*-induced antiapoptosis via attenuating CHOP-modulated apoptotic pathway in the GC cells.

### 3.6. Associations of ASPP2 and Clinicopathological Characteristics with Overall Survival (OS) in HPGC

**Figure 7:**
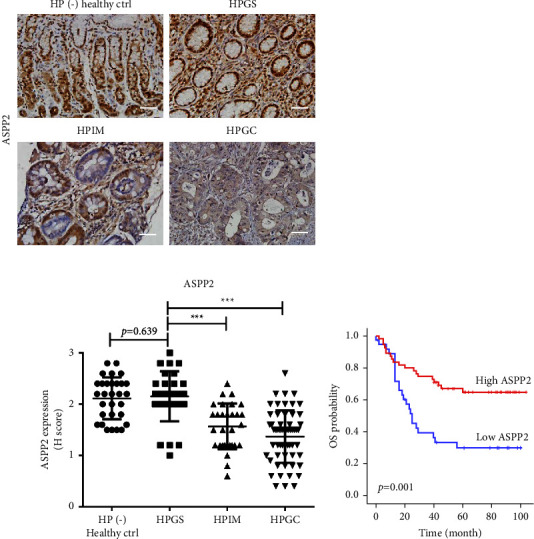
Decreased ASPP2 expression is associated with poor prognosis in HPGC. (a) Relative ASPP2 expression levels in gastric tissues from different stages of Correa's cascade, including HP (−) healthy control (*n* = 30), HPGS (*n* = 30), HPIM (*n* = 29), and HPGC (*n* = 57) by IHC. Upper, representative images were captured at 200x magnification. Scale bar, 50 *μ*m. Lower, relative quantitation of ASPP2 levels by *H* score ranking. The significance of differences in *H* score between two groups was compared using the nonparametric Mann–Whitney *U* test. (b) Kaplan–Meier OS curves for 92 HPGC patients stratified by ASPP2 expression were constructed using the Kaplan–Meier method and compared with the log-rank test. ^*∗∗∗*^*p* < 0.001.

To establish the ASPP2 level in gastric tissues, we examined histological patterns at different stages of Correa's cascade, including HP (−) healthy control (*n* = 30), HPGS (*n* = 30), HPIM (*n* = 29), and HPGC (*n* = 57) samples. As presented in Figure 7(a), expression of ASPP2 was markedly lower in gastric tissues from HPGC and HPIM stages relative to HPGS. No differences were observed between HPGS and HP (−) healthy control groups. The demographic and clinicopathological features and ASPP2 levels of all subjects analyzed in this study are shown in Supplementary Table 4. We consistently observed significantly lower ASPP2 expression in HPGC and HPIM gastric tissues compared to HPGS counterpart tissues. Moreover, ORs of demographic features and ASPP2 levels for HPGC were evaluated via logistic regression analysis. As shown in Table 1, univariate analysis revealed correlations of cigarette smoking, alcohol consumption, and ASPP2 levels with risk of HPGC. Multivariate logistic regression analysis confirmed that reduced expression of ASPP2 is associated with the higher risk of HPGC. We further assessed the HRs of demographic and clinicopathological features and ASPP2 levels in HPGC patients via Cox's proportional hazards analysis. As shown in Table 2, univariate analysis showed that tumor stage, invasive depth, nodal metastasis, metastasis, and ASPP2 levels were correlated with OS of HPGC. After adjustment for age, gender, tumor stage, invasive depth, nodal metastasis, and metastasis in multivariate analysis, metastasis and ASPP2 levels were correlated with OS of HPGC patients. The OS of HPGC patients in relation to ASPP2 expression was further examined using Kaplan–Meier survival curves. As shown in Figure 7(b), low expression of ASPP2 was associated with poor OS in HPGC patients. Based on the collective results, we proposed that reduced ASSP2 expression could serve as a novel independent prognostic predictor of HPGC.

## 4. Discussion

Limited knowledge exists regarding aberrantly expressed miRNAs and their targeted genes in GC [44]. MiR-21 is upregulated in many cancer types and promotes cancer progression by targeting several tumor suppressors [45]. Our experiments corroborated previous findings [17], showing that miR-21 promotes cell proliferation and metastasis and inhibits apoptosis to a significant extent in AGS cells. Moreover, miR-21 expression was remarkably increased following *H. pylori* infection in both the GC cells *in vitro* and gastric tumor tissues *in vivo*, analogous to earlier results [17, 46]. The activation of NF-*κ*B, increased IL-6 secretion, and enhanced expression of AP-1 and STAT3 by *H. pylori* are proposed mechanism for inducing miR-21 transcription [26]. NF-*κ*B has also been identified as an activating transcription factor for miR-21 in the GC cells [37, 47]. Our experiments demonstrated that NF-*κ*B regulates miR-21 expression by binding to its promoter region in *H. pylori*-infected cells, supporting a key role of miR-21 in HPGC development.

MiR-21 regulates tumor suppressors to promote cancer progression [15]. This study used proteomic approaches to identify 1896 potential downregulated proteins in miR-21-overexpressing AGS cells. The TargetScan program in miRSystem predicted 47 potential miR-21 targets [30]. Functional enrichment analysis revealed that these downregulated proteins were involved in cell death, survival, and cellular movement and growth. Previous studies demonstrated that miR-21 targets LZTFL1 in breast cancer cells, RASA1 in colon cancer cells, and KLF5 in hepatocellular carcinoma cells [48–50]. This suggests that miR-21 and its target genes could be the molecular targets of HPGC. To gain insight into the miR-21 target proteins, interaction network analysis was applied to identify protein clusters with similar functions [51]. As a result, 32 out of 47 miR-21 downregulated proteins related to enriched functions form the three clusters. Among these proteins, we identified five candidates of miR-21 targets, including PDCD4, ASPP2, DAXX, PIK3R1, and MAP3K1.

MiR-21 promotes growth and invasion by inhibiting PDCD4 in the GC cells [39]. It also induces cell migration and invasion in breast cancer cells by targeting PIK3R1 [52]. However, our study did not observe significant effects of miR-21 on PIK3R1 and MAP3K1 gene expression. *In silico* analyses suggest that DAXX contains miR-21 binding sites [53] and it promotes cell apoptosis and inhibits cell invasion in the GC cells [54]. Moreover, miR-21 suppresses ASPP2 to modulate cell apoptosis, proliferation, and metastasis in glioblastoma, hepatoblastoma, and lung cancer cells [53, 55, 56]. Among these candidates, ASPP2 was significantly upregulated following miR-21 inhibition in our experiments. Luciferase reporter assay further validated ASPP2 as a direct miR-21 target. In view of its identification as a miR-21 target and potential tumor suppressor activity, the functional importance of ASPP2 was further explored.

ASPP2 suppression is linked to cell growth and inhibition of apoptosis in hepatocellular carcinoma cells and leukemia cells [40, 57]. Here, ASPP2 was suppressed by miR-21 in *H. pylori*-infected GC cells. The *H. pylori*-derived cytotoxin-associated gene A disrupts the apoptotic function of ASPP2 in AGS cells [58, 59]. Our results showed that *H. pylori*-induced ASPP2 suppression enhanced antiapoptosis in the GC cells, which provides valuable clues for further investigating the signaling pathways involved in HPGC carcinogenesis. ASPP2 promotes apoptosis by binding to p53 and enhancing the transcription of proapoptotic genes, such as PUMA, PIG3, and Bax [60]. In addition to regulation of p53-dependent apoptosis, ASPP2 is implicated in apoptosis independent of p53 [40]. In most human cancer types, ASPP2 acts as a tumor suppressor and is often downregulated. For instance, ASPP2 knockdown suppresses p85*α* expression, leading to increased AKT phosphorylation and antiapoptosis in triple-negative breast cancer cells [61]. Downregulation of ASPP2 also confers resistance to apoptosis induced by 5-FU- and etoposide by promoting BECN1-dependent autophagy in hepatocellular carcinoma cells [62]. In our study, reduced ASPP2 expression promoted resistance to apoptosis in the *H. pylori*-infected GC cells via disruption of the CHOP-mediated apoptotic pathway. Based on protein interaction network and Co-IP assays, we identified CHOP as a direct mediator of ASPP2 proapoptotic activity in the *H. pylori*-infected GC cells. Previous study has shown that ASPP2 overexpression increases CHOP protein levels in hepatoma cells [63]. Here, ASPP2 silencing markedly reduced CHOP expression in the *H. pylori*-infected GC cells, suggesting ASPP2 involvement in CHOP degradation regulation. The critical finding indicated that loss of the ASPP2 and CHOP interaction promotes p300-mediated degradation of CHOP in the *H. pylori*-infected GC cells. CHOP is a transcriptional repressor via competing the promoter binding with C/EBP to inhibit C/EBP-mediated transcription of Bcl-2 and Bcl-xL [33, 34]. CHOP upregulates Bim expression, activating Bak and Bax to induce mitochondrial apoptosis [42]. In addition, CHOP and ATF5 could activate Noxa gene expression to induce apoptosis during stress [33]. Noxa, a BH3-only member of the Bcl-2 family, is required for apoptosis activation in response to ER stress. Our results demonstrate that CHOP mediates ASPP2-induced apoptosis by upregulating Noxa and Bak and suppressing Bcl-2 expression in the GC cells. Moreover, our study found that *H. pylori* initially promotes apoptosis in the GC cells, but later induces antiapoptosis through miR-21-mediated ASPP2 suppression, which disrupts CHOP-mediated apoptotic signaling by facilitating p300 dependent CHOP degradation in the GC cells. The overall data suggest that the *H. pylori*-infected host cells initiate alternative pathways to prevent apoptosis and promote GC development.

ASPP2 expression in *H. pylori-*positive cancer or precancerous tissues is reported to be lower than that in *H. pylori*-negative tissues [64]. In this study, ASPP2 was frequently downregulated in HPGC and HPIM compared with HPGS tissues, suggesting that *H. pylori* infection suppresses ASPP2 expression, leading to inhibition of apoptosis and promotion of cell proliferation. Our data support attenuation of ASPP2 expression as a mechanism to promote resistance to apoptosis in HPGC. Moreover, univariate OR analysis disclosed that lower ASPP2 expression is a risk factor of HPGC. Similarly, cigarette smoking and alcohol consumption were associated with the risk of HPGC, analogous to previous findings [4]. Although the incidence rate of GC varies by age and gender [65], ORs of these factors showed no significant associations in our study. Similarly, in multivariate OR analysis, lower ASPP2 level was significantly correlated with HPGC risk.

While ASPP2 shows prognostic value in human cancers [57, 66], its role in GC remains to be established. Data from our univariate HR analysis showed that lower ASPP2 expression is an indicator of poor prognosis in HPGC patients. Moreover, tumor stage, invasive depth, nodal metastasis, and metastasis had a significant influence on the OS of HPGC patients, which is consistent with previous results [67–70]. Although histological grade, tumor location, and Lauren classification are reported to be associated with GC prognosis [69, 71], these characteristics showed no significant associations in our study. Furthermore, in multivariate HR analysis, only metastasis and ASPP2 expression were correlated with the OS of HPGC patients. Previously, the significance of several clinicopathological features in GC prognosis was compared in different studies, with divergent findings [67–71]. This may be attributable to heterogeneous etiologies and mechanisms underlying GC development [4]. Lower ASPP2 expression was correlated with poor survival rate in an earlier study on esophageal squamous cell carcinoma patients [66]. Likewise, ASPP2 suppression was associated with high-risk disease and therapy failure in acute leukemia patients [57]. Here, we identified ASPP2 as a potential prognostic biomarker in the HPGC population.

The effectiveness of *H. pylori* eradication in HPGC prevention has achieved success. However, the efficacy is frequently compromised in those patients with precancerous lesions [72]. It indicates that other than *H. pylori* virulence factors, the aberrant host response underlying *H. pylori* infection is also important for developing new therapeutic approach of HPGC. In this study, we demonstrated dysregulation of ASPP2/CHOP pathway in response to *H. pylori*-induced miR-21 overexpression, which confers apoptosis resistance in host cells. Low expression of ASPP2 increases the occurrence of GC in the *H. pylori*-infected patients. Restoration of ASPP2 expression or activity may offer a breakthrough in developing therapeutic intervention against HPGC. Moreover, clinicopathological analyses suggested a predictive potential of lower ASPP2 in HPGC poor prognosis. In conclusion, our study provides both therapeutic and prognostic insights into developing precision medicine against HPGC.

## 5. Conclusions

**Figure 8:**
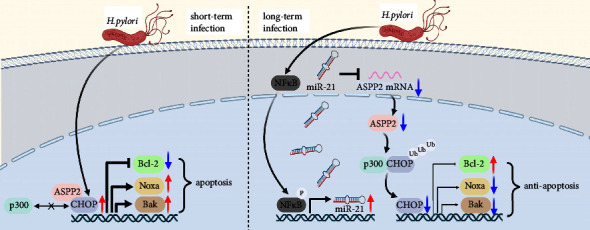
*H. pylori*-induced miR-21-mediated ASPP2 suppression confers resistance to apoptosis through inhibition of ASPP2/CHOP-mediated transcription of Noxa and Bak and suppression of Bcl-2 in the GC cells.

*H. pylori*-induced miR-21-mediated ASPP2 suppression confers resistance to apoptosis through inhibition of ASPP2/CHOP-mediated transcription of Noxa and Bak and suppression of Bcl-2 in gastric cancer cells (Figure 8). In addition, lower ASPP2 expression is associated with higher risk and poor prognosis in the *H. pylori*-induced gastric cancer patients.

## Figures and Tables

**Table 1:** ORs of demographic features and ASPP2 expression for HPGC via logistic regression analysis.

	Univariate analysis		Multivariate analysis	
	OR (95% CI)	*p* value	OR (95% CI)	*p* value
Age		0.078		0.120
Age <50 years	1		1	
Age ≥50 years	2.17 (0.91, 5.13)		2.19 (0.81, 5.94)	
Gender		0.061		0.985
Female	1		1	
Male	2.28 (0.98, 5.58)		1.01 (0.35, 2.94)	
Cigarette smoking		**<0.05^∗^**		0.210
Nonsmoker	1		1	
Smoker	6.48 (1.75, 42.10)		3.08 (0.61, 23.84)	
Alcohol consumption		**<0.05^∗^**		0.140
No	1		1	
Yes	7.95 (1.52, 146.69)		5.31 (0.80, 106.36)	
ASPP2 expression		**<0.05^∗^**		**<0.05^∗^**
High	1		1	
Low	4.37 (1.55, 15.73)		4.79 (1.58, 18.07)	

Note: ^∗^significant at the level of *α* = 0.05.

**Table 2:** HRs of demographic and clinicopathological features and ASPP2 expression in HPGC patients via Cox's proportional hazards analysis.

	Univariate analysis	
	HR (95% CI)	*p* value

Age		0.178
≥50 years vs. <50 years	1.69 (0.79, 3.66)	
Gender		0.732
Male vs. female	0.90 (0.49, 1.64)	
Cigarette smoking		0.273
Smoker vs. nonsmoker	1.45 (0.75, 2.83)	
Alcohol consumption		0.972
Yes vs. no	0.99 (0.45, 2.16)	
Tumor location		0.514
Proximal vs. distal	1.48 (0.46, 4.79)	
Lauren classification		0.730
Intestinal type vs. diffuse type	0.89 (0.46, 1.73)	
Tumor stage^#^		**<0.001^∗∗∗^**
III and IV vs. I and II	7.67 (3.53, 16.66)	
Histological grade		0.169
G3 and G4 vs. G1 and G2	1.72 (0.80, 3.70)	
Invasive depth		**<0.001^∗∗∗^**
T3 and T4 vs. T1 and T2	7.48 (2.93, 19.10)	
Nodal metastasis		**<0.001^∗∗∗^**
N2 and N3 vs. N0 and N1	3.16 (1.67, 6.01)	
Metastasis		**<0.001^∗∗∗^**
M1 vs. M0	18.60 (8.63, 40.11)	
ASPP2 expression		**<0.01^∗∗^**
Low vs. high	2.62 (1.42, 4.81)	

	Multivariate analysis^‡^	
	HR (95% CI)	*p* value

Tumor stage^#^		0.056
III and IV vs. I and II	3.87 (0.97, 15.48)	
Invasive depth		0.505
T3 and T4 vs. T1 and T2	1.65 (0.38, 7.10)	
Nodal metastasis		0.454
N2 and N3 vs. N0 and N1	0.73 (0.33, 1.65)	
Metastasis		**<0.001^∗∗∗^**
M1 vs. M0	9.08 (3.90, 21.10)	
ASPP2 expression		**<0.05^∗^**
High vs. low	2.15 (1.07, 4.31)	

*Note* :  ^‡^Adjusted for age, gender, tumor stage, invasive depth, nodal metastasis, and metastasis. ^#^Tumor stage was defined according to the 7^th^ edition of the American Joint Committee on Cancer Staging. ^∗^significant at the level of *α* = 0.05, ^∗∗^significant at the level of *α* = 0.01, and ^∗∗∗^significant at the level of *α* = 0.001.

## Data Availability

The data presented in this study are available on request from the corresponding author.

## Supplementary Materials

Supplementary Materials Supplementary Table 1: list of primer sequences, small nucleotide sequences, and specific target sequences of siRNA or shRNA. Supplementary Table 2: list of antibodies used in this study. Supplementary Table 3: top three enriched biological functions of 47 downregulated (fold change<0.7) proteins in AdmiR-21 AGS cells via IPA. Supplementary Table 4: demographic and clinicopathological features and ASPP2 levels in HPGC, HPIM, HPGS, and HP (−) healthy control groups. Supplementary Figure 1: *H. pylori* induces NF-*κ*B-mediated miR-21 overexpression in the GC cells. Supplementary Figure 2: upregulation of miR-21 impacts proliferation, migration, invasion, and apoptosis of the GC cells. Supplementary Figure 3: identification of potential miR-21 targets in the GC cells. Supplementary Figure 4: representative quantitative mass spectrum of potential miR-21 target, ASPP2. Supplementary Figure 5: ASPP2 plays a role in the GC cell apoptosis. Supplementary Figure 6: the effect of *H. pylori* infection on apoptosis in the GC cells. Supplementary Figure 7: CHOP is involved in ASPP2-mediated apoptosis via regulation of Noxa, Bak, and Bcl-2 in the GC cells after *H. pylori* infection.
