# Supplementary material for: miR-21 Targets ASPP2 to Inhibit Apoptosis via CHOP-Mediated Signaling in Helicobacter pylori-Infected Gastric Cancer Cells
Source: J Oncol. 2023 Jul 28;2023:6675265. doi: 10.1155/2023/6675265 (PMC10403333; doi:10.1155/2023/6675265)
Supplement: Supplementary Materials — Supplementary Table 1: list of primer sequences, small nucleotide sequences, and specific target sequences of siRNA or shRNA. Supplementary Table 2: list of antibodies used in this study. Supplementary Table 3: top three enriched biological functions of 47 downregulated (fold change<0.7) proteins in AdmiR-21 AGS cells via IPA. Supplementary Table 4: demographic and clinicopathological features and ASPP2 levels in HPGC, HPIM, HPGS, and HP (−) healthy control groups. Supplementary Figure 1: H. pylori induces NF-κB-mediated miR-21 overexpression in the GC cells. Supplementary Figure 2: upregulation of miR-21 impacts proliferation, migration, invasion, and apoptosis of the GC cells. Supplementary Figure 3: identification of potential miR-21 targets in the GC cells. Supplementary Figure 4: representative quantitative mass spectrum of potential miR-21 target, ASPP2. Supplementary Figure 5: ASPP2 plays a role in the GC cell apoptosis. Supplementary Figure 6: the effect of H. pylori infection on apoptosis in the GC cells. Supplementary Figure 7: CHOP is involved in ASPP2-mediated apoptosis via regulation of Noxa, Bak, and Bcl-2 in the GC cells after H. pylori infection. [file 6675265.f1.pdf]

Supplementary Materials and Methods

Cell proliferation, wound healing and invasion assays

Growth, migration and invasion abilities of AGS cells with or without miR-21 overexpression were analyzed. Cell proliferation was evaluated using MTT assay (Sigma-Aldrich; St Louis, MO, USA), cell migration assessed by the wound healing assay (Abcam, Cambridge, UK) and the invasive capability analyzed using a Matrigel-coated Boyden chamber assay (BD Biosciences, San Jose, CA, USA).

Bioinformatic analysis

To obtain an ASPP2-mediated apoptotic network, protein-protein interactions among ASPP2 interactants and apoptosis-associated proteins were explored via ingenuity pathway analysis (IPA, Ingenuity Systems, Redwood City, CA, USA; http://www.ingenuity.com) and a graphical representation of the linkage network among the molecules mapped. Genes or gene products are presented as nodes and the biological relationship between two nodes represented by edges. All edges are supported by at least one literature reference and canonical information stored in the Ingenuity Pathways Knowledge Base. Those with connection numbers ≥10 were considered potential mediators in the ASPP2-associated apoptotic network.

Supplementary Table 1. List of primer sequences, small nucleotide sequences and specific target sequences of siRNA or shRNA

Method	Product	Sequence
**RT**	RT-miR-21 primer	GTCGTATCCATGGCAGGGTCCGAGGTATTCGCCATGGATACGACTCAACA
	RT-U6 primer	GTCGTATCCATGGCAGGGTCCGAGGTATTCGCCATGGATACGACAAAATATGGAACGCTT
**qPCR**	miR-21 primer	F: CCGCGGTAGCTTATCAGACTGA
	U6 primer	F: GTGCTCGCTTCGGCAGCACA
	universal reverse primer	R: TGGCAGGGTCCGAGGT
	ASPP2 primer	F: GAAGACTCGGTGAGCATGCG<br>R: GCGATACGCTCTGAGCCAGT
	CHOP primer	F: GCGCATGAAGGAGAAAGAAC

	R: CCAATTGTTCATGCTTGGTG
Noxa primer	F: GCTGGAAGTCGAGTGTGCTA<br>R: CCTGAGCAGAAGAGTTTGGA
Bak primer	F: TGGTCACCTTACCTCTGCA<br>R: TCAAACAGGCTGGTGGCAAT
Bcl-2 primer	F: ATGTGTGTGGAGACCGTCAA<br>R: GCCGTACAGTTCCACAAAGG
Bim primer	F: ACAGAGCCACAAGACAGGAG<br>R: CCATTGCACTGAGATAGTGGTTG
Bax primer	F: CCCGAGAGGTCTTTTTCCGAG<br>R: CCAGCCCATGATGGTTCTGAT

	PUMA primer	F: GACGACCTCAACGCACAGTA<br>R: AGGAGTCCCATGATGAGATTGT
	DR5 primer	F: AAGACCCTTGTGCTCGTTGT<br>R: AGGTGGACACAATCCCTCTG
	Bcl-xL primer	F: GCTGGTGGTTGACTTTC<br>R: GGATGGGTTGCCATTGA
	Mcl-1 primer	F: TGCTTCGGAAACTGGACATTAAA<br>R: ATGGGTCATCACTCGAGAAAAAG
	GAPDH primer	F: ATGGGGAAGGTGAAGGTCG<br>R: GGGTCATTGATGGCAACAATATC
**ChIP-qPCR**	miR-21 promoter primer	F: GGAGTGGATGGGTTCTGCCTTA<br>R: CAAGGTGGATTGCATCGAGG

	Noxa<br>promoter primer	F: CCA AGA GAT GCT GGA ATC TGG<br>R: GGC TCC CTA GAA GTG CTT AG
	Bak<br>promoter primer	F: GAGATGGAGTCTTGCACTGTCAC<br>R: CCT GTA ATC CCA GCT AGT TGG
	Bcl-2<br>promoter primer	F: CAGCGAAGGTGCCGGGGCTCC<br>R: GGA TGA CTG CTA CGA AGT TCT C
**small nucleotide transfection assay**	miR-21 mimic	UAGCUUAUCAGACUGAUGUUGAAACAUCAGUCUGAUAAGCUAUU
	anti-miR-21	UCAACAUCAGUCUGAUAAGCUA
**shRNA knockdown assay**	shASPP2 target sequence	AGTGTTTGAATAAGCGTAATTC

**siRNA knockdown assay**	siCHOP target sequence	CAAUUGUUCAUGCUUGGUGUU
	sip300 target sequence	GCACAAAUGUCUAGUUCUUTT

Supplementary Table 2. List of antibodies used in this study

Antibodies	Suppliers
**Phosphorylated p65 (p-p65, 3033)**	Cell Signaling Technology, Danvers, MA, USA
**P65 (8242)**	Cell Signaling Technology, Danvers, MA, USA
**β-actin (sc-47778)**	Santa Cruz Biotechnology, Santa Cruz, CA, USA
**ASPP2 (sc-53861)**	Santa Cruz Biotechnology, Santa Cruz, CA, USA
**CHOP (2895)**	Cell Signaling Technology, Danvers, MA, USA
**Control IgG (GTX 213110-01)**	Genetex, Irvine, CA, USA
**P300 (sc-48343)**	Santa Cruz Biotechnology, Santa Cruz, CA, USA
**Ubiquitin (04-263)**	Merck Millipore, Burlington, MA, USA
**Noxa (14766)**	Cell Signaling Technology, Danvers, MA, USA
**Bak (12105)**	Cell Signaling Technology, Danvers, MA, USA
**Bcl-2 (15071)**	Cell Signaling Technology, Danvers, MA, USA
**Caspase 9 (9508)**	Cell Signaling Technology, Danvers, MA, USA
**Cleaved caspase 3 (9664)**	Cell Signaling Technology, Danvers, MA, USA
**PARP (9532)**	Cell Signaling Technology, Danvers, MA, USA

Supplementary Table 3. Top three enriched biological functions of 47 downregulated (fold change<0.7) proteins in AdmiR-21 AGS cells via IPA

Functions	*p* value	Molecules
**Cell death and survival**	1.91E-07	ADNP, ARHGEF12, ASF1A, **ASPP2**, **DAXX**, DDAH1, DNM1L, GATAD2B, KLF5, LZTFL1, **MAP3K1**, MTMR12, NFAT5, PAN3, PCBP1, **PDCD4**, PFKM, PGM1, PHF6, PHLDB1, **PIK3R1**, PPP3CA, RASA1, SSFA2, STAT3, TBL1XR1, TNRC6B, TRIM33, VSNL1, ZFP36L2
**Cellular movement**	6.53E-05	ARHGEF12, **ASPP2**, **DAXX**, KLF5, **MAP3K1**, NFAT5, **PDCD4**, PHF6, **PIK3R1**, PPP3CA, PTPN9, RASA1, STAT3, VSNL1, ZNF217
**Cellular growth and proliferation**	2.41E-03	ADNP, **ASPP2**, **DAXX**, DNM1L, LZTFL1, **MAP3K1**, **PDCD4**, PFKM, **PIK3R1**, TBL1XR1, TRIM33, ZNF217

Supplementary Table 4. Demographic and clinicopathological features and ASPP2 levels in HPGC, HPIM, HPGS and HP (-) healthy control groups

	HP (-) healthy control (n=30)	HPGS (n=30)	HPIM (n=29)	HPGC (n=92)
**Age (year), mean±SD**	42.47±12.93	51.33±10.64	60.96±12.43	60.03 ± 13.55
**Gender (male/female)**	13/17	10/20	12/17	49/43
**Cigarette smoking (yes/no/NA[ǂ])**	6/22/2	2/28/0	5/17/7	25/54/13
**Alcohol consumption (yes/no/NA[ǂ])**	5/23/2	1/29/0	6/16/7	17/62/13
**Tumor location (proximal/distal)**				5/87
**Lauren classification (intestinal/diffuse/mixed/unclassified)**				33/42/9/8
**Tumor Stage[#] (I/II/III/IV)**				24/22/27/19
**Histological grade (G1/G2/G3/G4)**				5/20/57/10
**Invasive depth (T1/T2/T3/T4)**				25/10/5/52
**Nodal metastasis (N0/N1/N2/N3)**				33/15/17/27
**Metastasis (M0/M1)**				74/18

**ASPP2 levels (high/low)**	30/0	26/4	18/11†	55/37*

‡Missing data

#Tumor stage was defined according to the 7th edition of the American Joint Committee on Cancer staging

†*p*<0.05 compared with HPGS using Mann-Whitney test

**p*<0.01 compared with HPGS using Mann-Whitney test

Supplementary figure

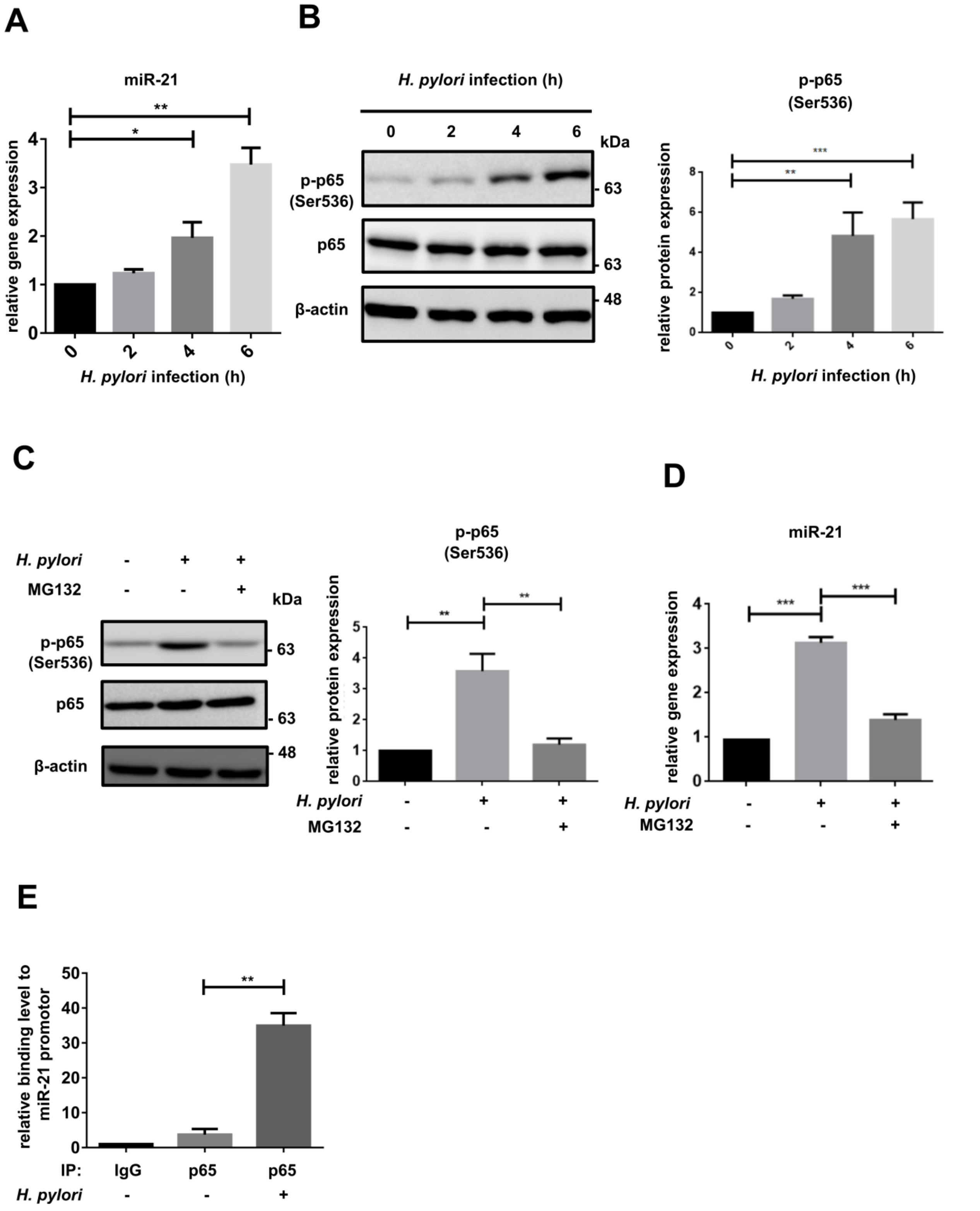

Supplementary Figure 1. *H. pylori* induces NF-κB-mediated miR-21 overexpression in GC cells. (**A-B**) AGS cells were incubated with *H. pylori* at 30 MOI for the indicated time. (A) Relative miR-21 levels in the cell lysates were analyzed by qPCR. (B) NF-κB activation in the cell lysates were

analyzed via immunoblotting. **(C-D)** AGS cells treated with 10μM MG132 or DMSO for 1.5 h were incubated with *H. pylori* at 30 MOI or PBS for 6 hours. (C) NF-κB inhibition in the cell lysates were measured by immunoblotting. (D) Relative miR-21 levels in the cell lysates were analyzed by qPCR. (**E**) Chromatin of AGS cells incubated with *H. pylori* at 30 MOI or PBS for 6 hours were immunoprecipitated with IgG or p65 antibodies immobilized on protein A beads for 16 hours. ChIP complex was retrieved and analyzed for relative p65 binding level to promoters of miR-21 using qPCR. All the data were presented as mean ± SD of 3 replicates. *$p<0.05$; **$p<0.01$; ***$p<0.001$.

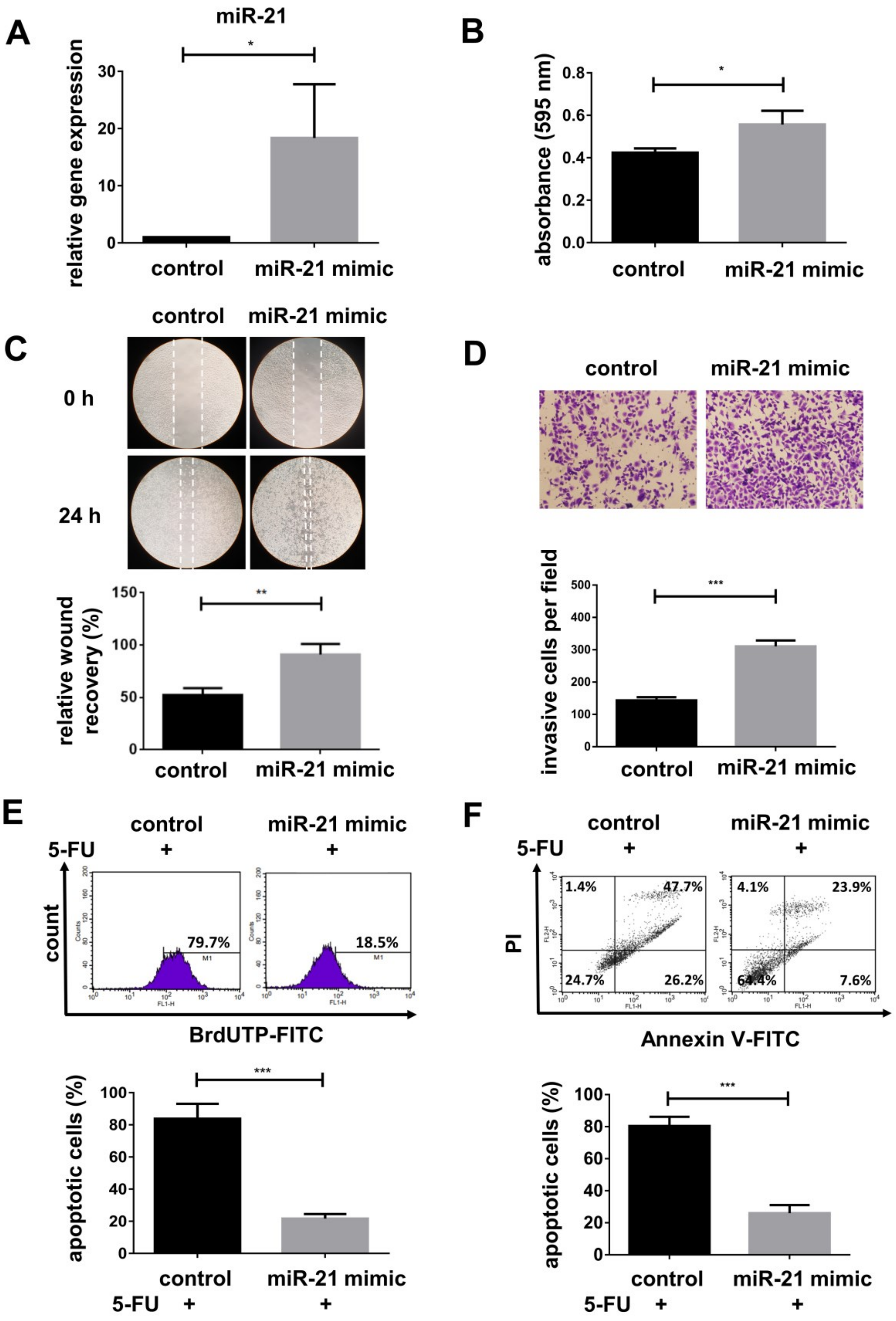

Supplementary Figure 2. Upregulation of miR-21 impacts proliferation, migration, invasion and apoptosis of GC cells. **(A)** AGS cells were transfected with control or miR-21 mimic oligonucleotides. miR-21 levels were measured by qPCR. **(B-D)** AGS cells were transfected with

control or miR-21 mimic oligonucleotides. Cell proliferation, migration and invasion abilities were evaluated via (B) MTT, (C) wound healing and (D) Matrigel-coated Boyden chamber assays. (**E-F**) AGS cells transfected with control or miR-21 mimic oligonucleotides were stimulated with 2.5 mM 5-FU for 16 hours. (E) The percentage of apoptotic cells were analyzed by TUNEL assay. (F) The percentage of early and late apoptotic cells were assessed via Annexin V/ PI assay. All the data were presented as mean ± SD of 3 replicates. *p<0.05; **p<0.01; ***p<0.001.

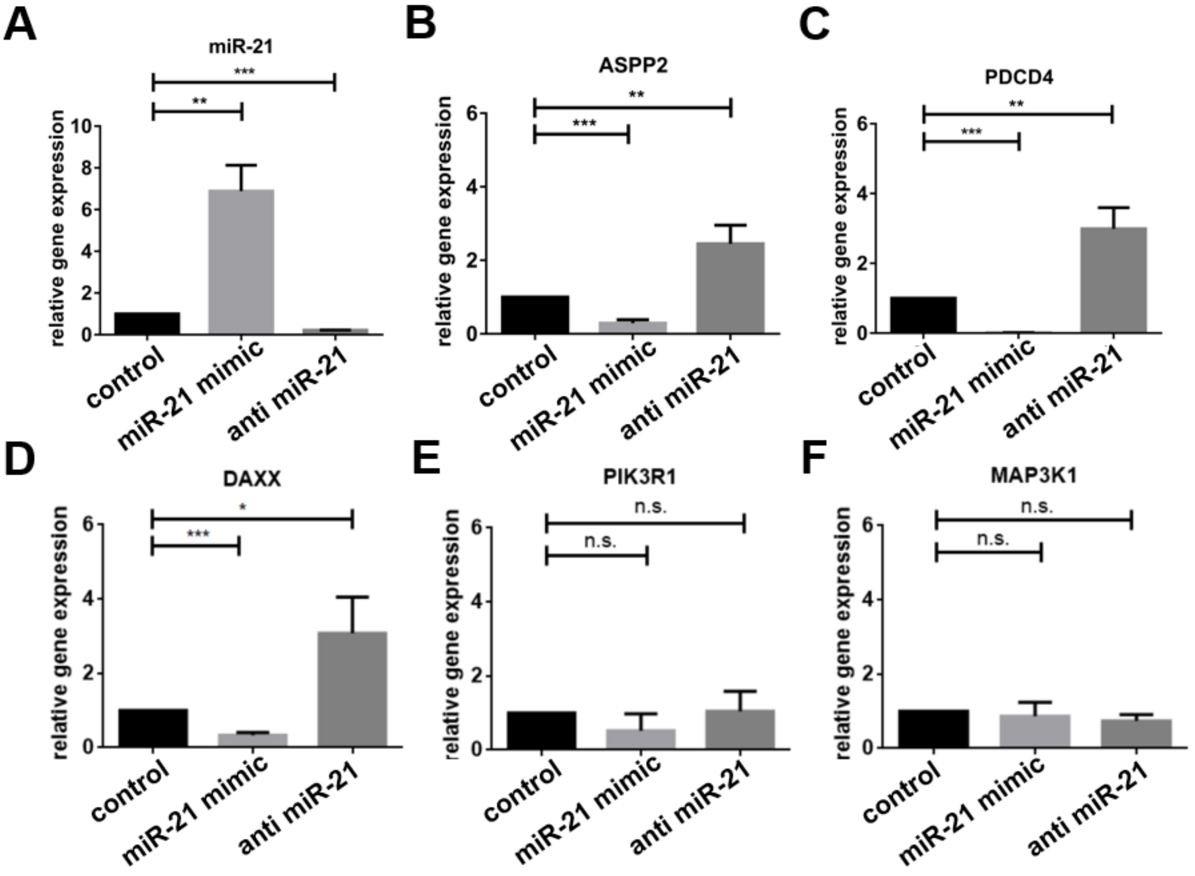

Supplementary Figure 3. Identification of potential miR-21 targets in GC cells. AGS cells were transfected with control, miR-21 mimic or anti-miR-21 oligonucleotides. **(A)** Relative miR-21 levels and relative mRNA levels of **(B)** ASPP2, **(C)** PDCD4, **(D)** DAXX, **(E)** PIK3R1, and **(F)** MAP3K1 were measured by qPCR. All the data were presented as mean ± SD of 3 replicates. n.s. non-significance; **p*<0.05; ***p*<0.01; ****p*<0.001.

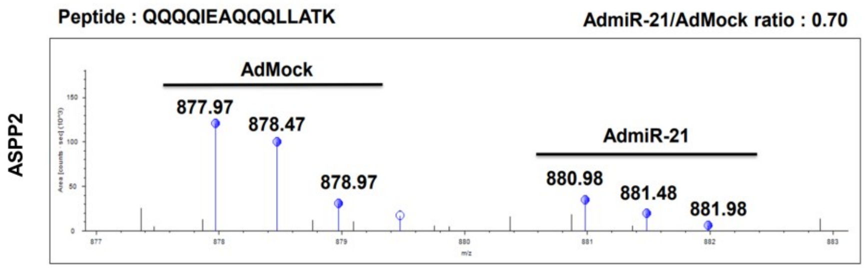

Supplementary Figure 4. Representative quantitative mass spectrum of potential miR-21 target, ASPP2. Proteins were extracted from AdMock and AdmiR-21 AGS cells and digested with trypsin. Peptide mixture was fractionated via off-line high-performance liquid chromatography and analyzed with an LTQ-Orbitrap Velos hybrid mass spectrometer. Acquired spectra were processed, analyzed and quantified using Proteome Discoverer software with the Mascot search engine against the Swiss-Prot *Homo sapiens* protein database.

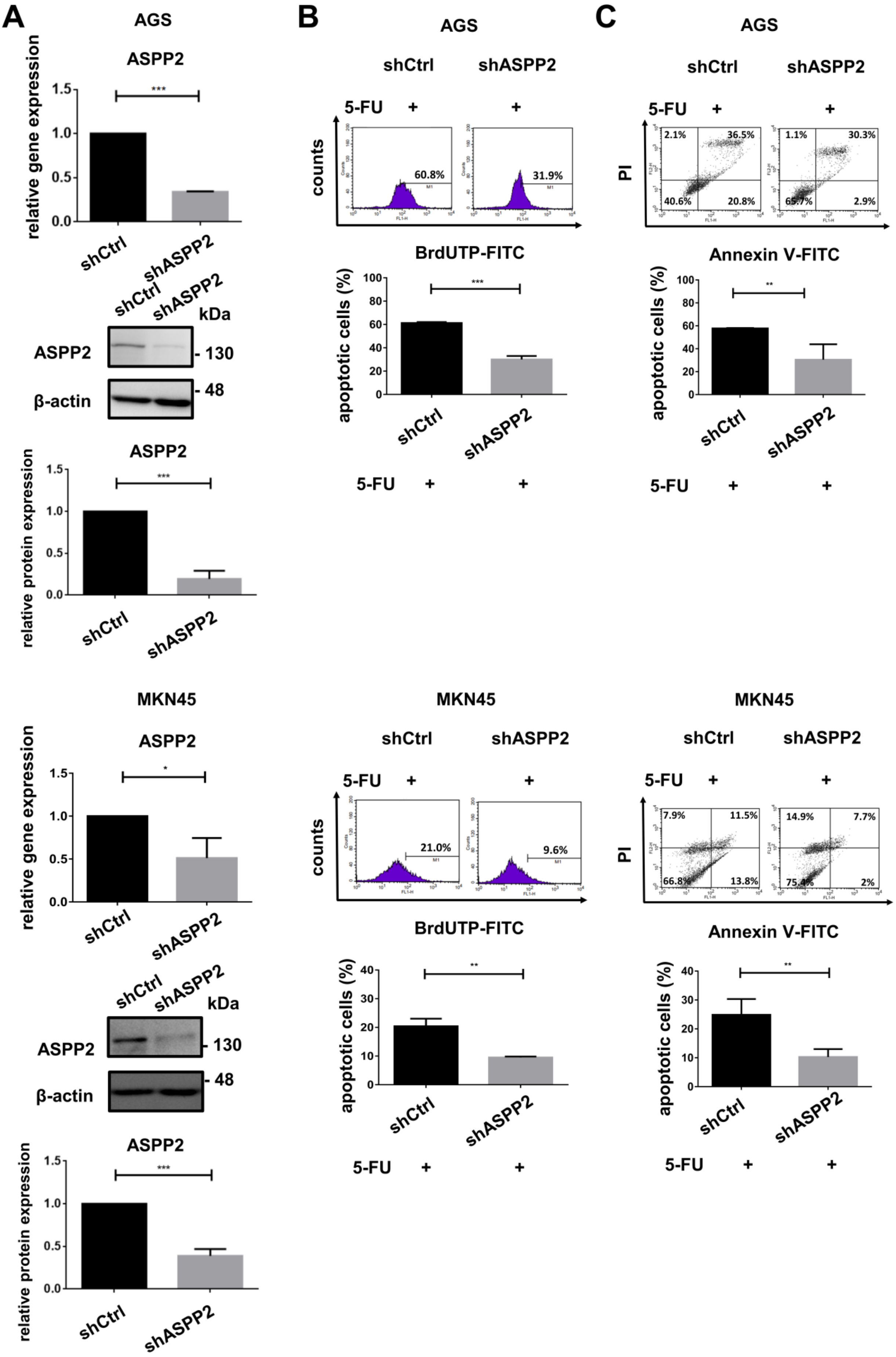
A
AGS
ASPP2
relative gene expression
shCtrl
shASPP2
ASPP2
β-actin
kDa
130
48
relative protein expression
B
AGS
shCtrl
shASPP2
5-FU
+
counts
60.8%
31.9%
BrdUTP-FITC
apoptotic cells (%)
C
PI
2.1%
36.5%
40.6%
20.8%
1.1%
30.3%
65.7%
2.9%
Annexin V-FITC
MKN45
21.0%
9.6%
7.9%
11.5%
66.8%
13.8%
14.9%
7.7%
75.4%
2%

Supplementary Figure 5. ASPP2 plays a role in GC cell apoptosis. (A) AGS and MKN45 cells were infected with lentivirus expressing control or ASPP2 shRNA for 72 hours to establish shCtrl and shASPP2 cells. The relative ASPP2 mRNA and protein levels in cell lysates were analyzed by qPCR and immunoblotting. (**B-C**) shCtrl and shASPP2 AGS and MKN45 cells were stimulated with 2.5 and 7.5 mM 5-FU for 16 hours. (B) The percentage of apoptotic cells were analyzed by TUNEL assay. (C) The percentage of early and late apoptotic cells were assessed via Annexin V/ PI assay. All the data were presented as mean ± SD of 3 replicates. *p<0.05; **p<0.01; ***p<0.001.

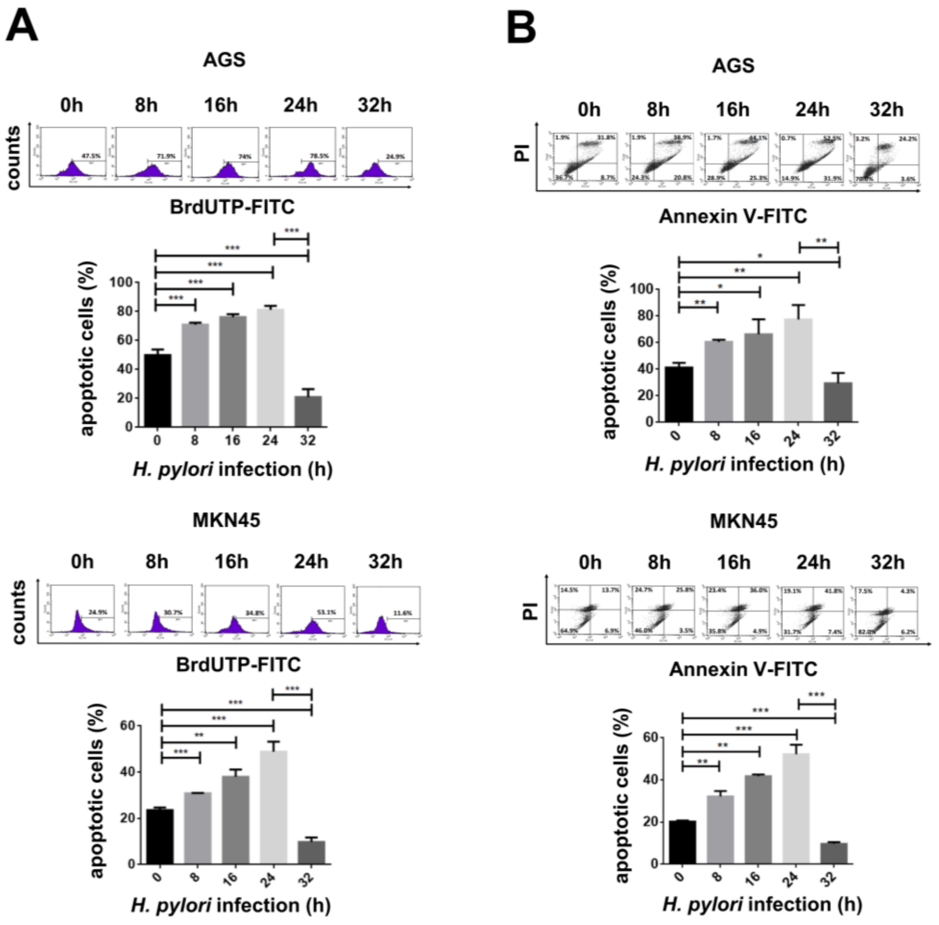

Supplementary Figure 6. The effect of *H. pylori* infection on apoptosis in GC cells (**A, B**) AGS and MKN45 cells were incubated with *H. pylori* at 30 and 90 MOI for the indicated time. (A) The percentage of apoptotic cells were analyzed by TUNEL assay. (B) The percentage of early and late apoptotic cells were assessed via Annexin V/ PI assay. All the data were presented as mean ± SD of 3 replicates. *p<0.05; **p<0.01; ***p<0.001.

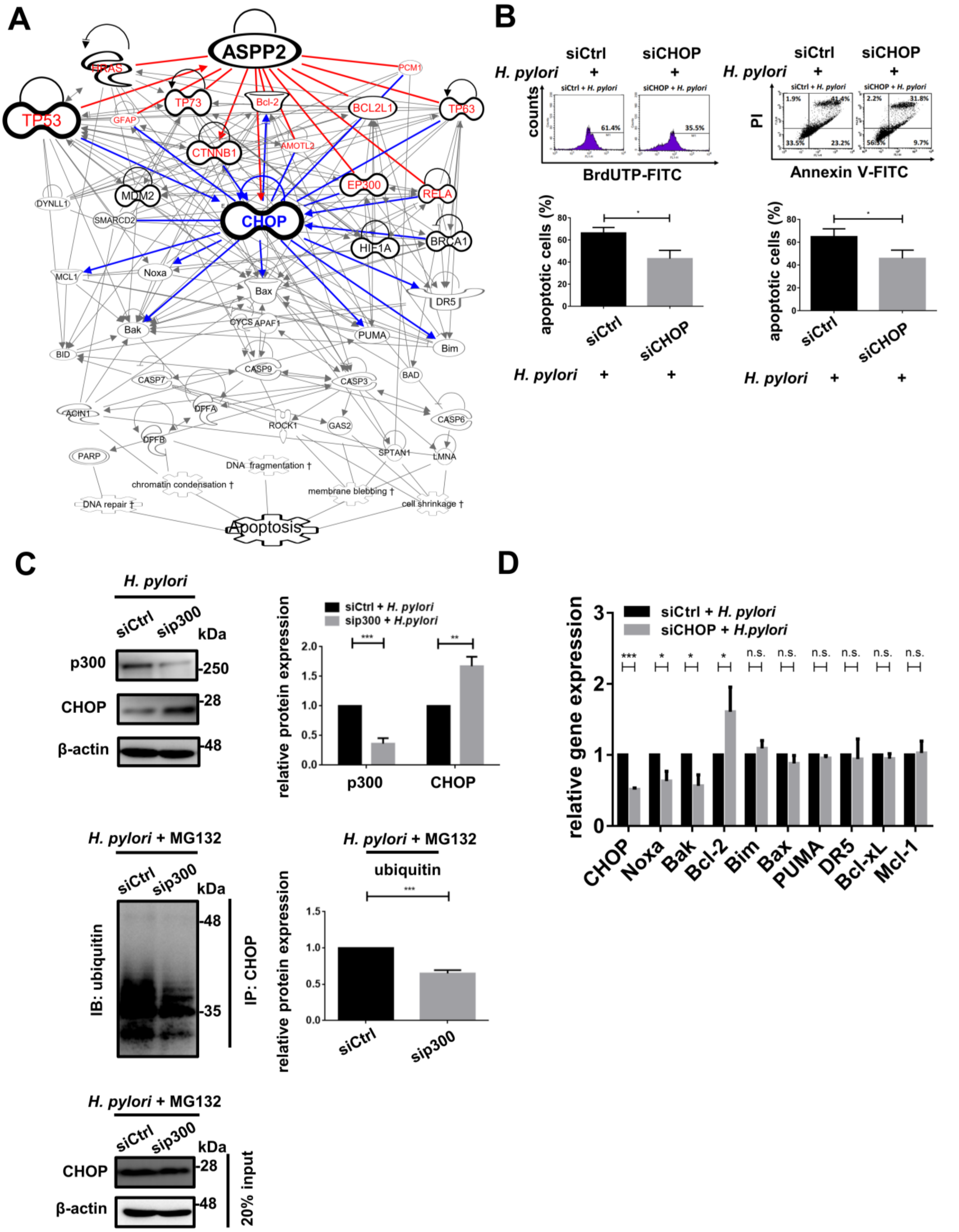

Supplementary Figure 7. CHOP is involved in ASPP2-meidated apoptosis via regulation of Noxa, Bak and Bcl-2 in GC cells after *H. pylori* infection. (A) Identification of CHOP as potential

mediator of ASPP2-mediated apoptosis via IPA. Protein-protein interactions network among ASPP2 interactants and apoptosis-associated proteins were constructed. Among these proteins, CHOP was identified as a potential mediator of ASPP2-mediated apoptosis network showing 20 interaction linkage. **(B)** AGS cells transfected with siCtrl and siCHOP were incubated with *H. pylori* at 30 MOI for 8 h. Left, the percentage of apoptotic cells were analyzed by TUNEL assay. Right, the percentage of early and late apoptotic cells were assessed via Annexin V/ PI assay. **(C)** AGS cells transfected with siCtrl and sip300 were incubated with *H. pylori* at 30 MOI for for 8 h. Cell lysates were analyzed via immunoblotting (upper). Cell lysates of siCtrl and sip300 AGS cells incubated with *H. pylori* at 30 MOI for 8 h followed by 10 μM MG132 for 3 h were incubated with antibodies against CHOP immobilized on protein A beads for 16 hours. The Co-IP complex (middle) and 20% input (lower) were measured via immunoblotting. **(D)** AGS cells transfected with siCtrl and siCHOP were incubated with *H. pylori* at 30 MOI for 8 h. The mRNA levels of apoptotic factors in cell lysates were analyzed by qPCR. All the data were presented as mean ± SD of 3 replicates. n.s. non-significance; *p<0.05; **p<0.01; ***p<0.001.
